# Improved consolidated bioprocessing for itaconic acid production by simultaneous optimization of cellulase and metabolic pathway of *Neurospora crassa*

**DOI:** 10.1186/s13068-024-02505-5

**Published:** 2024-04-29

**Authors:** Chen Zhao, Jiajia Zhao, Jingjing Han, Yaojie Mei, Hao Fang

**Affiliations:** 1 College of Life Sciences, Northwest A&F University, 22 Xinong Road, Yangling, 712100 Shaanxi China; 2 Biomass Energy Center for Arid and Semi-Arid Lands, Northwest A&F University, Yangling, 712100 Shaanxi China; 3 The Second Department of Vaccine, Lanzhou Institute of Biological Products Co., Ltd., Lanzhou, 730046 China; 4 Key Laboratory of Biomass Chemical Engineering of Ministry of Education, College of Chemical and Biological Engineering, Zhejiang University, Hangzhou, 310027 China; 5 ZJU-Hangzhou Global Scientific and Technological Innovation Center, Zhejiang University, 733 Jianshe 3rd Road, Hangzhou, 311200 Zhejiang China

**Keywords:** Cellulose, Consolidated bioprocessing, *Neurospora crassa*, Cellulase, Itaconic acid

## Abstract

Lignocellulose was directly used in itaconic acid production by a model filamentous fungus *Neurospora crassa*. The promoters of two clock control genes and cellobiohydrolase 1 gene were selected for heterologous genes expression by evaluating different types of promoters. The effect of overexpression of different cellulase was compared, and it was found that expression of cellobiohydrolase 2 from *Trichoderma reesei* increased the filter paper activity by 2 times, the cellobiohydrolase activity by 4.5 times, and that the itaconic acid titer was also significantly improved. A bidirectional cis-aconitic acid accumulation strategy was established by constructing the reverse glyoxylate shunt and expressing the transporter MTTA, which increased itaconic acid production to 637.2 mg/L. The simultaneous optimization of cellulase and metabolic pathway was more conducive to the improvement of cellulase activity than that of cellulase alone, so as to further increase itaconic acid production. Finally, through the combination of fermentation by optimized strains and medium optimization, the titers of itaconic acid using Avicel and corn stover as substrate were 1165.1 mg/L and 871.3 mg/L, respectively. The results prove the potential of the consolidated bioprocessing that directly converts lignocellulose to itaconic acid by a model cellulase synthesizing strain.

**Supplementary Information:**

The online version contains supplementary material available at 10.1186/s13068-024-02505-5.

## Introduction

Through unique photosynthesis, plants capture atmospheric carbon to produce lignocellulose with an annual production of about 170 billion tons, which is the most abundant renewable on Earth [1]. Lignocellulose has been considered as one of the most potential production raw materials to replace fossil resources due to its non-competitive, renewable, low-cost and carbon–neutral properties [2]. Lignocellulosic carbohydrates consist mainly of cellulose and hemicellulose, which make up 60–80% of the total dry mass of the cell wall and, therefore, play an important role in the bio-refining system [3]. Many microorganisms secrete cellulases to degrade cellulose and hemicellulose, and the sugars produced can be further converted into a range of products. The process of using microorganisms to complete the conversion of lignocellulose to product in the same system is called consolidated bioprocessing (CBP), which utilizes a single vessel/bioreactor to obtain valuable products from biomass in one step, and has the greatest potential for cost reduction compared to traditional separated hydrolysis and fermentation (SHF) and simultaneous saccharification and fermentation (SSF) [4, 5]. However, the challenge is related to it. At present, the efficiency of CBP is very low, and it is difficult to carry out industrial application [6]. While with the development of synthetic biology and metabolic engineering, the construction of efficient cell factories has attracted more and more attention, bringing new hope to the development of CBP [7–9].

The complex structure of lignocellulose requires microorganisms to secrete multiple cellulases to break it down into monomer forms, which are then fermented or converted into valuable compounds. This makes it challenging to complete the CBP process using a single microorganism. Filamentous fungi have great potential for breaking down lignocellulosic biomass, and most commercial cellulases and other biomass hydrolases are produced by them [10, 11], which makes it more possible to establish an efficient single microbial CBP system. However, the challenges are also obvious, including efficient expression of various heterologous genes in the construction pathway, effective compatibility of the new pathway with the existing pathways in the cell to maximize the yield, and the balance between the secretion of cellulase and the synthesis of products [12].

In a previous study, we constructed the itaconic acid synthesis pathway in *Neurospora crassa* to enable initial itaconic acid synthesis [13]. *N. crassa* is a filamentous fungus with rapid growth, a wide spectrum of carbon sources, and the ability to secrete a large amount of cellulase, showing potential for CBP [14–16]. In addition, it has a clear background as a model biomolecule and is convenient for gene manipulation [17, 18]. Itaconic acid is a five-carbon unsaturated dicarboxylic acid that can be used as a sustainable, non-toxic alternative to many petroleum-derived monomers in the production of commercially viable synthetic resins, coatings and polymers [19]. Products include lubricants, plasticizers, adhesives, detergent additives, anti-rust additives and gelation accelerators, etc. [20]. The world market of itaconic acid is expected to reach 170,000 tons per year by 2025, with sales of about $255 million [21]. Currently, itaconic acid is produced by natural strains, such as *Aspergillus terreus* and *Ustilago maydis*. Through metabolic engineering, *Aspergillus niger*, *Escherichia coli*, *Saccharomyces cerevisiae* and *Yarrowia lipolytica* have also been able to synthesize itaconic acid, mostly using simple substrates such as glucose in the fermentation process [8]. The main factor in itaconic acid production is the cost problem, as it competes with petroleum-derived platform chemicals. Therefore, it is necessary to design microbial cell factories with efficient fermentation routes to produce high levels of itaconic acid on low-cost substrates [8]. Some studies used cheap cellulose as a substrate, but required the addition of commercial cellulase or fermentation broth containing cellulase [22–24]. We expect that by modifying existing chassis microorganisms that can synthesize large quantities of cellulase enable the synthesis of itaconic acid, and increase its yield.

In this study, promoters of *N. crassa* used for heterologous gene expression were evaluated to screen for strong promoters that can be subsequently used for the expression of multiple genes. The weakness of cellulase system in the synthesis of itaconic acid by CBP was identified and overcome by the expression of various cellulase components, respectively. On the other hand, metabolic engineering optimization of itaconic acid synthesis was carried out around how to increase the concentration of the precursor cis-aconitic acid. Finally, the titer of itaconic acid was further improved by combining cellulase optimization and metabolic pathway optimization, and itaconic acid synthesis was attempted using corn stover as substrate in minimum medium. This study showed an example of how a cellulase-producing strain could be engineered to synthesize itaconic acid from cellulose.

## Materials and methods

### Media and culture conditions

Many studies have detailed media and procedures for *N. crassa* growth and molecular manipulation [25, 26]. For culture purpose, the cells first were grown on an agar medium containing 2% (w/v) sucrose and 1 × Vogel’s salts at 30 ℃ for 2 days under dark conditions and 6 days under light conditions. When a large number of conidia were formed, they were collected and made into conidia solution. The conidia (1 × 10^6^/mL final concentration) were then fed into seed medium containing 2% (w/v) sucrose and 1 × Vogel’s salts and cultured at 28 ℃ under dark conditions for 40 h. The hyphae were collected into fermentation medium containing 2% (w/v) Avicel and 1 × Vogel’s salts for 48 to 120 h at 28 ℃ under dark conditions. All shaker speeds were 220 rpm.

Corn stover was collected from Lianyungang City, Jiangsu Province, China. It was ground to pass through 80-mesh sieve and then dried to constant weight before use. When corn stover was used as the substrate, the corresponding medium for different cultures was as follows (every 50 mL medium): C1–only 0.5 g corn stover was added; C2–0.5 g corn stover with inorganic salt mixture; C3–C2 added 0.025 g ammonium sulfate; C4–C2 added 0.25 g ammonium sulfate; C5–C3 added trace elements mixture; C6–C3 added 0.3% talcum powder (MgSiO_3_, particle size less than 350 mesh, purchased from Sigma); A3–0.5 g Avicel with inorganic salts mixture and 0.025 g ammonium sulfate; A5–A3 added trace element mixture; A6–A5 added 0.3% talcum powder. Among them, inorganic salts mixture included: KH_2_PO_4_ 0.1 g, MgSO_4_ 0.015 g, CaCl_2_ 0.015 g, and trace elements mixture included: FeSO_4_·7H_2_O 0.25 mg, ZnSO_4_·7H_2_O 0.07 mg, MnSO_4_·4H_2_O 0.08 mg, CoCl_2_ 0.1 mg. All fermentations were carried out in 250 mL flasks filled with 50 mL of media.

### Construction of plasmids and strains

The *cad1* gene encoding cis-aconitic acid decarboxylase (GenBank ID: AB326105.1) was derived from *A. terreus* [13]. The genome of *N. crassa* wild-type FGSC2489 was used as a template for promoter amplification, and a sequence of about 1.0 kb upstream of the alternative gene was selected as the promoter. The promoters tested were *Pccg-1*, *Peas*, *Pgh11-2*, *Pcbh-1*, *Pgh6-2*, *Ptef-1*, *Pgpd* and *Ppda*. They were derived from clock control genes *ccg-1*, *ccg-2*, cellulase GH11-2, CBH1, GH6-2, α-elongation factor, glyceraldehyde-3-phosphate dehydrogenase and pyruvate decarboxylase, respectively. The promoter and optimized *cad1* gene were linked at *Xba I* restriction endonuclease sites, and connected into the plasmids pMF272 by *Not I* and *Pac I* sites (Additional file 1: Fig. S1A). Then, eight different promoter plasmids were obtained for expressing *cad1* gene (Additional file 1: Table S1). When cellulase was overexpressed or heterogeneously expressed, the gene fragments *cbh1*, *gh6-2*, *gh5-1*, *asbga*, *trcbh2* and terminator *TtrpC* were inserted into the *Xba I*-*EcoR I* sites by seamless cloning method, respectively, and the vector PMF-272-Pccg1-CBH1, PMF-272-Pccg1-GH6-2, PMF-272-Pccg1-GH5-1, PMF-272-Pccg1-AsBGA, and PMF-272-Pccg1-TrCBH2 were constructed (Additional file 1: Fig. S1B). When CAD and cellulase were co-expressed, the expression cassettes of the two genes were connected in series and inserted into the plasmid *Not I*-*EcoR I* sites (Additional file 1: Fig. S1C). Cellulase genes of *cbh1*, *gh6-2*, *gh5-1*, and *gh3-4* were derived from the wild type of *N. crassa.* The *asbga* gene encoding β-glucosidase (GenBank ID: KJ739789) and the *trcbh2* gene encoding cellobiohydrolase 2 (GenBank ID: M16190) were derived from *Aspergillus niger* and *Trichoderma reesei*, respectively. Malate thiokinase (MTK), composed of SucC (GenBank ID: 3104196) and SucD (GenBank ID: 3104195) subunits, was derived from *Methylococcus capsulatus*. To verify its expression effect in *N.crassa*, *sucC* and *sucD* genes were linked with a linker “GGCGGCTCGGGCGGCGGCTCCGGCGGCGGCAGCGGC”, and then inserted into pMF272 plasmid *Xba I*-*Pac I* locus (Additional file 1: Fig. S1D) to obtain plasmid pMF-272-Pccg1-MTK. Malyl-CoA lyase (MCL, GenBank ID: GU320612.1) derived from *Rhodobacter sphaeroides* was connected with promoter *Peas* and terminator *TtrpC*, and inserted into pMF-272-Pccg1-CAD *EcoR I* site to obtain plasmid pMF-272-Pccg1-CAD-Pes-MCL (Additional file 1: Fig. S1E). MTK expression cassette, hygromycin expression cassette and upstream as well as downstream homologous arms were connected and inserted into plasmid pUC19–MTK–HPH *Kpn I* site to obtain plasmid pUC19–MTK–HPH (Additional file 1: Fig. S1F). The homologous arms were designed according to the downstream sequence of MCL terminator *TtrpC* with a length of 1.5 kb. Cis-aconitic acid transporter MTTA (GenBank ID: HG423568.1), derived from *Aspergillus terreus*, was connected with promoter *Pcbh1* and terminator *TtrpC*, and inserted into pMF-272-Pccg1-CAD in the same way to obtain pMF-272-Pccg1-CAD-Pcbh1-MTTA. Similarly, The MTTA expression cassette and MCL expression cassette, MTTA expression cassette and TrCBH2 expression cassette were inserted into pMF-272-Pccg1-CAD to obtain pMF-272-Pccg1-CAD-Pcbh1-MTTA-Pes-MCL (Additional file 1: Fig. S1G) and pMF-272-Pccg1-CAD-Pcbh1-MTTA-Pcbh1-TrCBH2 (Additional file 1: Fig. S1H), respectively. The above pMF-272-related plasmids were transformed into *N.crassa* FGSC 9720 competent cells by electroporation, respectively, to obtain corresponding transformants [26]. The target strains were screened by the transformation plasmid to compensate for histidine defects. The plasmid pUC19–MTK–HPH was transformed into the strains containing pMF-272-Pccg1-CAD-Pes-MCL and pMF-272-Pccg1-CAD-Pcbh1-MTTA-Pes-MCL, respectively. The positive strains were selected by hygromycin resistance and named *N. crassa* PMF–CAD–rGS and *N. crassa* PMF–CAD–MTA–rGS, respectively. All heterologous genes were optimized according to the codon bias of *N. crassa* and synthesized by Sangon Biotech (Shanghai, China). The selected heterokaryons were further processed by the method described by Ebbole et al. to obtain homokaryons [27]. Primers, plasmids and strains used in this study are shown in Additional file 1: Tables S1–S6. The construction process of the main strains (Additional file 1: Figs. S2–S6), as well as the synthetic gene sequences, are shown in Additional file 1.

### Quantitative PCR (qPCR) analysis

The mycelia in the fermentation process were collected by vacuum filtration, dried by filter paper and stored in liquid nitrogen. After grinding the mycelia, RNA was extracted with an RNA extraction purification kit. The cDNA extraction system (20 μL) consisted of 1 μL total RNA, 4 μL 5*TransScript Uni All-in-One Super Mix, and 1 μL gDNA Remover. PCR reaction was performed at 50 ℃ for 5 min to amplify single-strand cDNA, and then at 85 ℃ for 5 s to inactivate the enzyme. Single strand cDNA was used for quantitative detection. The reaction system (20 μL) consisted of 1 μL cDNA, 0.4 μL forward/reverse primers each, and 2 × perfectStart Green qPCR SuperMix 10 μL. Cycling parameters were set as follows: 94 ℃ for 30 s; 42 cycles of 5 s at 94 ℃, 15 s at 55 ℃, and extension for 10 s at 72 ℃. Primers used are shown in Additional file 1: Table S7. The qPCR data were calculated using the 2^−△△Ct^ method to obtain the relative fold change of the target groups. The gene of *gh6-2*, *gh3-4* or *actin* was used as the internal reference gene.

### Metabolite analysis

For intracellular TCA cycle related metabolites analysis, the mycelium was quickly collected by filtration, frozen with liquid nitrogen and then ground. Pre-cooled methanol: chloroform (3:1) solvent along with internal standard adonitol was added to the ground cell mixture. The samples were vortexed for 3 min and then centrifuged at 4℃. The supernatant was concentrated to dryness using a vacuum concentrator. For metabolite derivation, 40 μL of methoxyamination hydrochloride (20 mg/mL in pyridine) was first added to the dry extract and incubated at 80 ℃ for 30 min, and then, 50 μL of BSTFA reagent (1% TMCS) was added and incubated at 70 ℃ for 1.5 h. Subsequently, 50 μL of BSTFA regent (1% TMCS, v/v) was added and incubated at 70 ℃ for 1.5 h. Then, the samples were analyzed by Agilent 7890 gas chromatograph coupled with a time-of-flight mass spectrometer. The column used for all analyses was a DB-5MS capillary column. Helium, as the carrier gas, had a flow rate of 3 mL/min at the front inlet and 1 mL/min in the column. The temperatures of inlet, interface, and ion source were 280, 280 and 250 °C, respectively. One microliter aliquot of the sample was injected into the GC in splitless mode, with the oven temperatures programmed at 50 °C for 1 min. Thereafter, the temperature was raised with a gradient of 10 °C/min until 310 °C, and held at this temperature for 8 min. The mass spectrometer was operated in full-scan mode (start after 6.2 min, mass range 50–550 amu). The processing of raw data was according to the procedures established by Dunn et al. [28].

For extracellular itaconic acid analysis, the supernatant of fermentation liquid was taken directly and detected by a Shimadzu LC-20A HPLC system equipped with a Bio-Rad Aminex HPX-87H column. The detection methods were detailed in our previous research [13].

### Fluorescence analysis

For screening and imaging, mycelia were placed onto a microscope slide and imaged using a dual-channel photomultiplier tube with a confocal system on a Leica DMi8 microscope (CLSD-2SS, Thorlabs). Samples were excited at 488 nm with a 1.4 NA × 100 oil immersion objective. Fluorescence was detected between 497 and 531 nm.

### Enzyme activity analysis

Filter paper (FP) activity, cellobiohydrolase (CB) activity and β-glucosidase (BG) activity were determined according to the method of Ghose [29]. Extracellular soluble protein content was determined by Bradford method. Specific filter paper activity (SFPA), specific cellobiohydrolase (SCBA) activity and specific β-glucosidase (SBGA) activity represented the cellulase activity per mg extracellular protein, respectively. The relative FP activity, relative CB activity or relative BG activity was the ratio of SFPA, SCBA or SBGA between the tested strain and the control strain.

After the mycelium was collected by filtration, the removal of cell wall was based on the method reported by Galazka et al. [30]. Lysis of protoplasts was performed by using a glass homogenizer (2 mL), and the specific method was referred to Isakova et al. [31]. The resulting homogenate was further centrifuged (4 ℃, 16,000×*g*, 10 min) to obtain the supernatant. The soluble protein of the supernatant was determined by Bradford method. The methods for assaying intracellular MTK–MCL conjugation enzyme activity were modified from Mainguet et al. [32]. Briefly, the reaction system (500 μL) contained 50 mM Tris–Cl (pH 7.5), 5 mM MgCl_2_, 2 mM phenylhydrazine, 10 mM malic acid, 2 mM ATP, 1 mM CoA and above supernatant containing 8 μg soluble protein. The reaction was performed at 37 °C and the formation of glyoxylate–phenylhydrazone was recorded at 324 nm using a spectrophotometer. The enzyme activity was calculated according to the glyoxylate standard curve (0, 10, 20, 30, 40 mM glyoxylate).

## Results

### Promoter evaluation

**Fig. 1:**
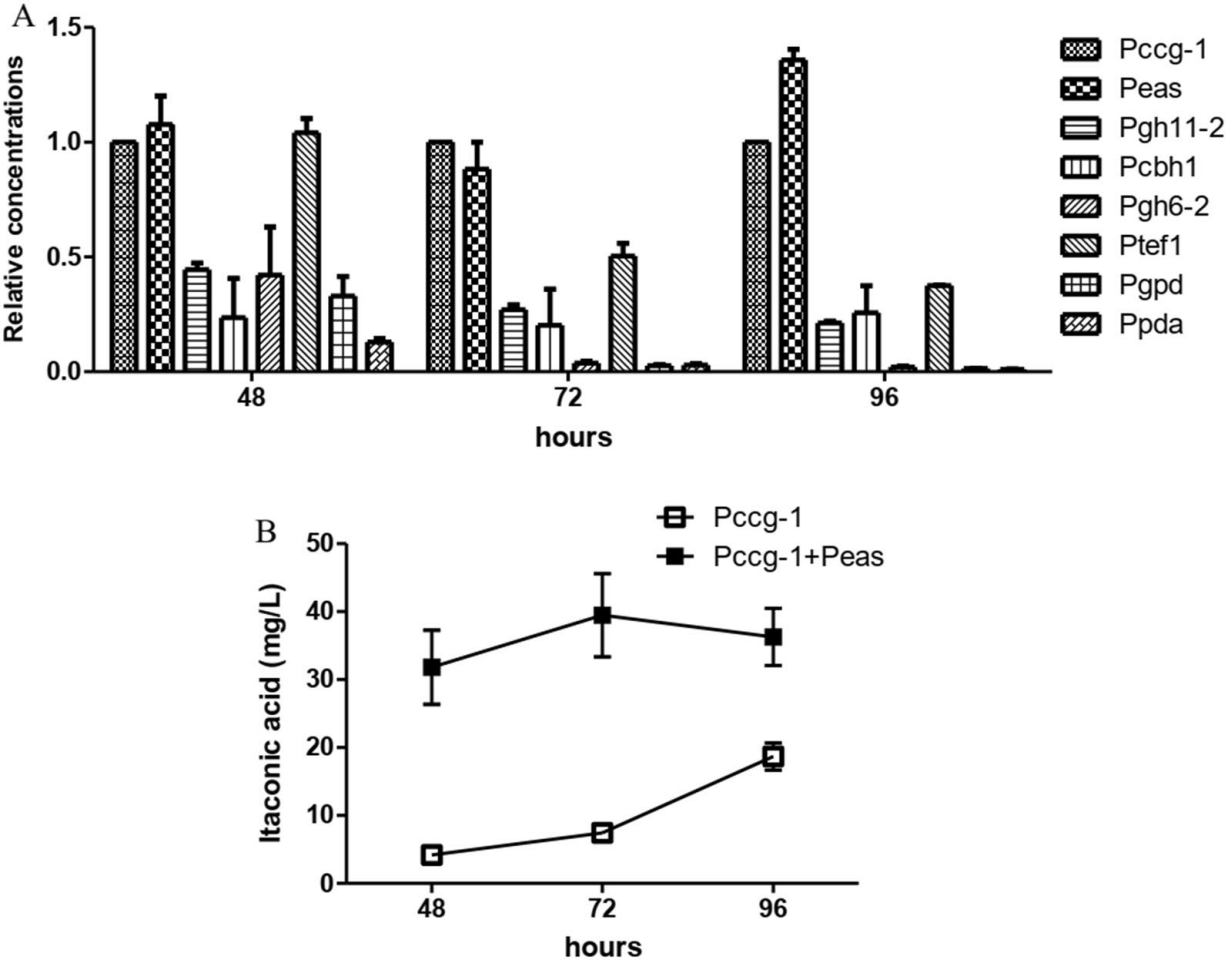
Production of itaconic acid at 48, 72, 96 h using different promoters expressing CAD. **A** Single-copy expression; **B** two-copy expression

To achieve high expression of both endogenous and exogenous genes, a strong promoter is needed to drive robust transcription. In the experiments, promoter *Peas*, *Pgh11-2*, *Pcbh-1*, *Pgh6-2*, *Ptef-1*, *Pgpd*, *Ppda* and *Pccg-1* were selected to express *cis*-aconite decarboxylase (CAD), and eight types of transformants were obtained. Itaconic acid was synthesized using Avicel as substrate, and the results are shown in Fig. 1A, where the result of *Pccg-1* is used as reference. The synthesis of itaconic acid by CAD using *Pccg-1* as promoter is shown in Fig. 1B. The results show that all 8 kinds of promoters could induce CAD synthesis of itaconic acid at 48 h. Compared with *Pccg-1*, the expression of CAD with *Peas* produced the most itaconic acid, which was 1.4 times higher than that with *Pccg-1*. The relative concentration of *Ptef-1* was comparable to that of *Pccg-1* and *Peas* at 48 h, but it decreased after 72 h. The production of cellulase promotors *Pgh11-2* and *Pgh6-2* was only about 50% of that of *Pccg-1* at 48 h. The production of promoter *Pgpd* and *Ppda*, which are associated with glucose metabolism, was also lower. The promoter *Pccg-1* and *Pea* were used to express CAD, respectively, and the two expression cassettes were expressed in series. Figure 1B shows that the titer of itaconic acid reached 31.8 mg/L at 48 h, and the highest titer was 39.5 mg/L at 72 h, which was 2.1 times that of CAD expressed with *Pccg-1* as the promoter alone. It can be seen that CAD copy number is directly related to itaconic acid production. *N. crassa ccg-1* is a clock control gene and is also considered to be a glucose suppressor gene [33]. The promoter of this gene can be significantly induced on non-glucose medium, such as sodium acetate as carbon source, and has been used in the expression of several proteins in *N. crassa*, such as GFP [34], RNase A [35], etc. *Peas* is allelic to *ccg-2* that can be expressed in large quantities without carbon source or cellulose [36]. It has not been reported as a strong promoter for heterologous protein expression. Since the content of glucose in the fermentation broth during CBP was very low, the cellulose could induce these two promoters and realize the expression of CAD. GH11-2, CBH-1 and GH6-2 are cellulases, however, when induced by Avicel, the effect of cellulase promoter was inferior to that of *Pccg-1* and *Peas*. The evaluation of promoters provided the selection of multiple strong promoters for the subsequent expression of multiple heterologous genes in *N. crassa*.

### Effect of expression of different cellulase components on cellulase and itaconic acid production

**Fig. 2:**
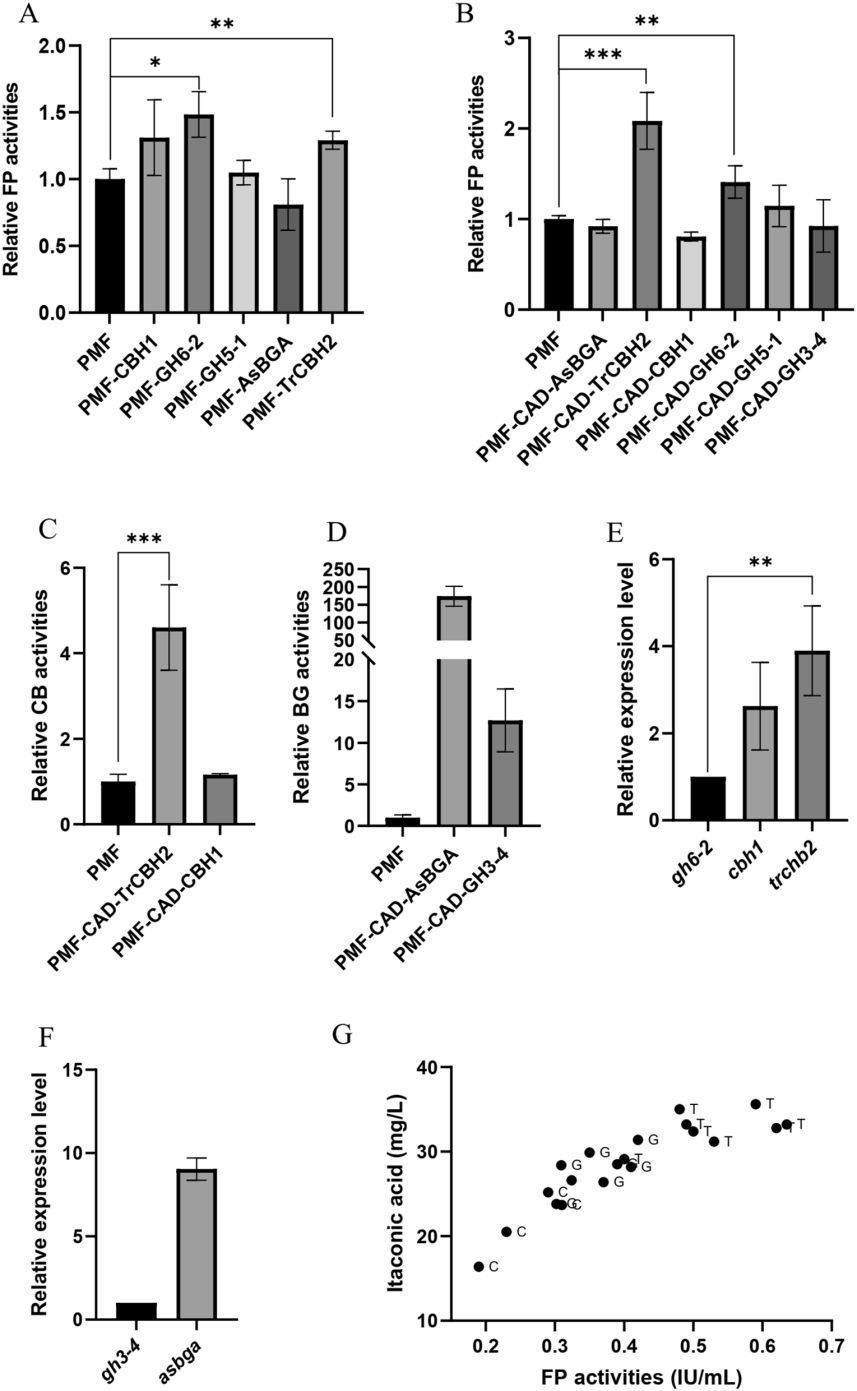
**A** Relative filter paper (FP) activities of the strain expressing the major cellulase component using *Pccg-1* as the promoter. PMF is a control strain supplemented with PMF-272; **B** relative FP activities of the strain expressing the major cellulase component and CAD using *Pcbh1* and *Pccg-1* as the promoter, respectively; **C** relative cellobiohydrolase (CB) activities of the strain PMF–CAD–TrCBH2 and PMF–CAD–CBH1; **D** relative β-glucosidase (BG) activities of the strain PMF–CAD–AsBGA and PMF–CAD–GH3-4; **E** expression levels of *cbh1* and *trcbh2* relative to *gh6-2* of the strain PMF–CAD–TrCBH2; **F** expression level of *asbga* relative to *gh3-4* of the strain PMF–CAD–AsBGA; **G** FP activities and itaconic acid titers of different recombinant strains. C: the control strain, G: the strain PMF–CAD–GH6-2, T: the strain PMF–CAD–TrCBH2. The above fermentation parameters were calculated according to the fermentation parameters of the 3 transformants. Two or three parallel groups were set for each transformants. The data of low FP activity and itaconic acid titer due to mycelial agglomeration were removed during calculation. The transcription level of cellulase was measured by the optimum transformant induced by Avicel for 4 h. *T* tests were conducted to evaluate statistical significance at *p* < 0.05 (*), *p* < 0.01 (**) and *p* < 0.001 (***)

In the process of cellulose conversion to itaconic acid, glucose and cellobiose were undetectable in the medium, indicating that the secretion of cellulase is a rate-limiting step. There are many kinds of cellulases, and the overall level of cellulases can be improved by finding and overcoming the weakness. Therefore, we overexpressed major cellulases in the *N. crassa,* respectively, to investigate their effects on cellulase activity and itaconic acid production. The cellulase components cellobiohydrolase1 (CBH1), cellobiohydrolase 2 (GH6-2) and endoglucanase (GH5-1) of *N. crassa* were overexpressed by using *Pccg-1* as promoter. The FP activity of strain PMF–GH6-2 expressing GH6-2 was 1.37 and 1.48 times higher than that of control PMF at 48 h and 72 h, respectively (Fig. 2A). At the same time, expressions were derived from the CBH2 of *T. reesei* (TrCBH2) and β-glucosidase of *Aspergillus niger* (AsBGA), respectively. It can be seen that the FP activity of strain PMF–TrCBH2 was significantly increased. To further verify the role of cellulase components in the itaconic acid producing strain, the CAD and the cellulase component were co-expressed, where the *Pcbh1* promoter of *N. crassa* was used to express cellulase components. Using the genes of *gh6-2* and *cbh1* of *N. crassa* as reference, the expression level of *trcbh2* was 3.9 times higher than that of *gh6-2* at 4 h of Avicel induction (Fig. 2E), and exceeded the expression level of *cbh1*, the highest cellulase component in *N. crassa*. It is obvious that the FP activity of PMF–CAD–TrCBH2 had the largest increase (Fig. 2B), which was twice that of the control, and the corresponding cellobiohydrolase activity was also significantly increased, which was 4.5 times that of the control (Fig. 2C). Compared with GH6-2 of *N. crassa*, the expression of *T. reesei* CBH2 had a more significant effect on the cellulase activity. On the other hand, the β-glucosidase activities of PMF–CAD–AsBGA and PMF–CAD–GH3-4 were analyzed, and it was found that both of them were significantly increased at 48 h (Fig. 2D). The expression level of *A. niger* β-glucosidase in PMF–CAD–AsBGA was nearly nine times higher than that of GH3-4 in *N. crassa* (Fig. 2F)*.* However, the FP activity was not high, indicating that the system of the strain did not lack β-glucosidase. The FP activity and itaconic acid titer of different transformants were tested, and the overall trend was that their itaconic acid titer increased with the increase of FP activity (Fig. 2G). The increase of TrCBH2 was the largest, and the highest itaconic acid titer was 35.6 mg/L. These results indicate that overexpression of cellobiohydrolase 2 (TrCBH or GH6-2) could increase cellulase activity and itaconic acid production. Above research shows that the cellulase system optimization could promote more carbon source to synthesize itaconic acid.

### Metabolic regulation promotes itaconic acid synthesis

**Fig. 3:**
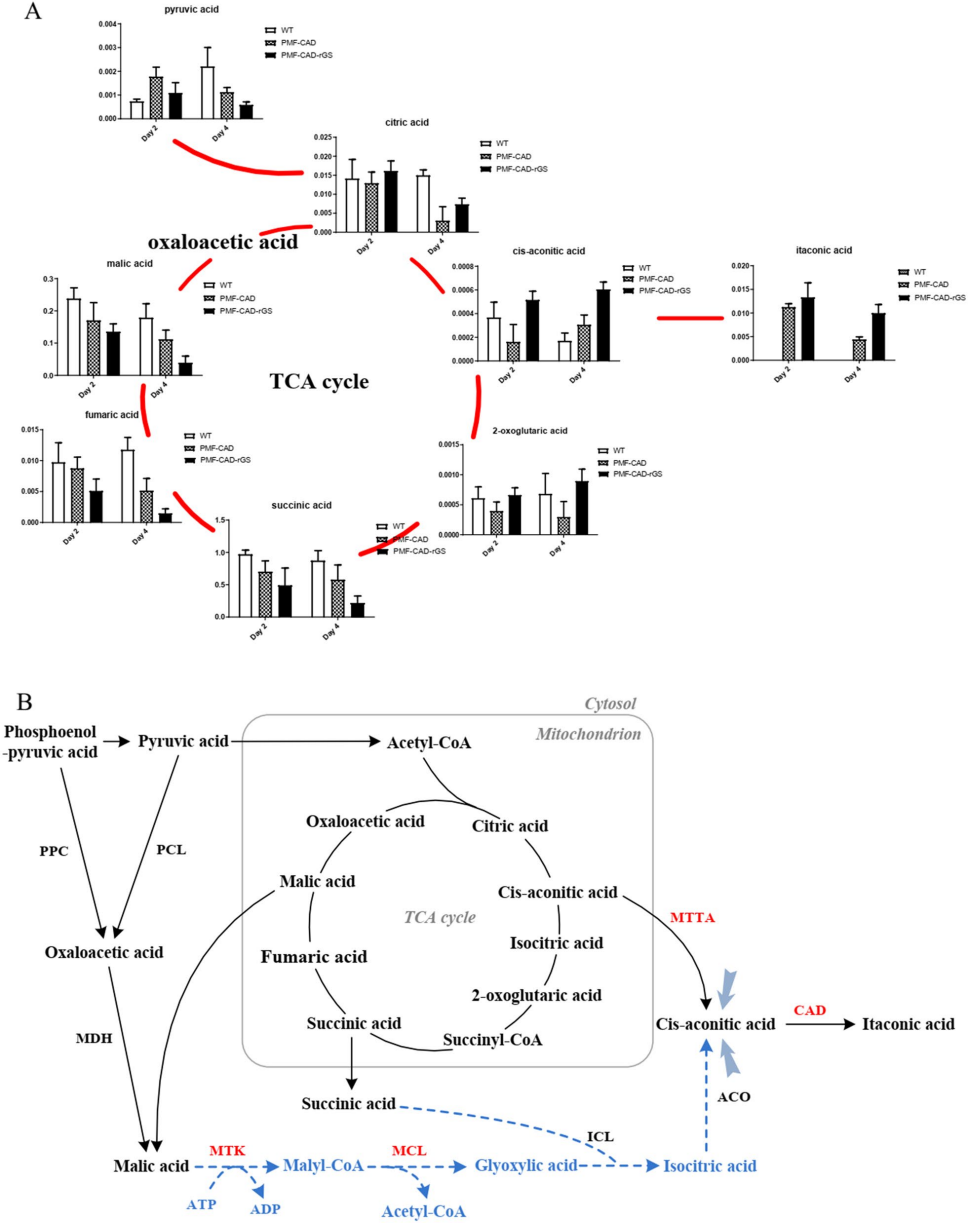
**A** Comparison of relative concentrations of intermediate metabolites in the TCA cycle. **B** Synthetic pathway of itaconic acid. Blue dotted line: cis-aconitic acid was synthesized from malic acid through the reverse glyoxylate shunt constructed in this study; Red font: heterologous expression enzyme/transporter. *MTK* malate thiokinase, *MCL* malyl-CoA lyase, *MTTA* cis-aconitic acid transporter, *CAD* cis-aconitic acid decarboxylase, *PPC* phosphoenol–pyruvate carboxykinase, *PCL* pyruvate carboxylase, *MDH* malate dehydrogenase, *ICL* isocitrate lyase, *ACO* aconitate hydratase

The analysis of TCA cycle intermediate metabolites of wild-type strain and CAD-expressing strains showed that the relative concentrations of malic acid and succinic acid were significantly higher than other metabolites (Fig. 3A). For example, the relative concentration of succinic acid in wild-type strain was 0.97 on the second day, and was still 0.70 after the expression of CAD, while the intracellular concentration of itaconic acid was only 0.01. Therefore, we hypothesized that if the carbon source could return from the reducing end of the TCA cycle to the oxidizing end, the amount of itaconic acid precursors could be increased. Therefore, in this study, a reverse glyoxylate shunt (rGS) was established, allowing malic acid to convert glyoxylic acid in two steps, and glyoxylic acid as well as succinic acid to form isocitric acid, thereby increasing the content of the precursor cis-aconitic acid (Fig. 3B).

**Fig. 4:**
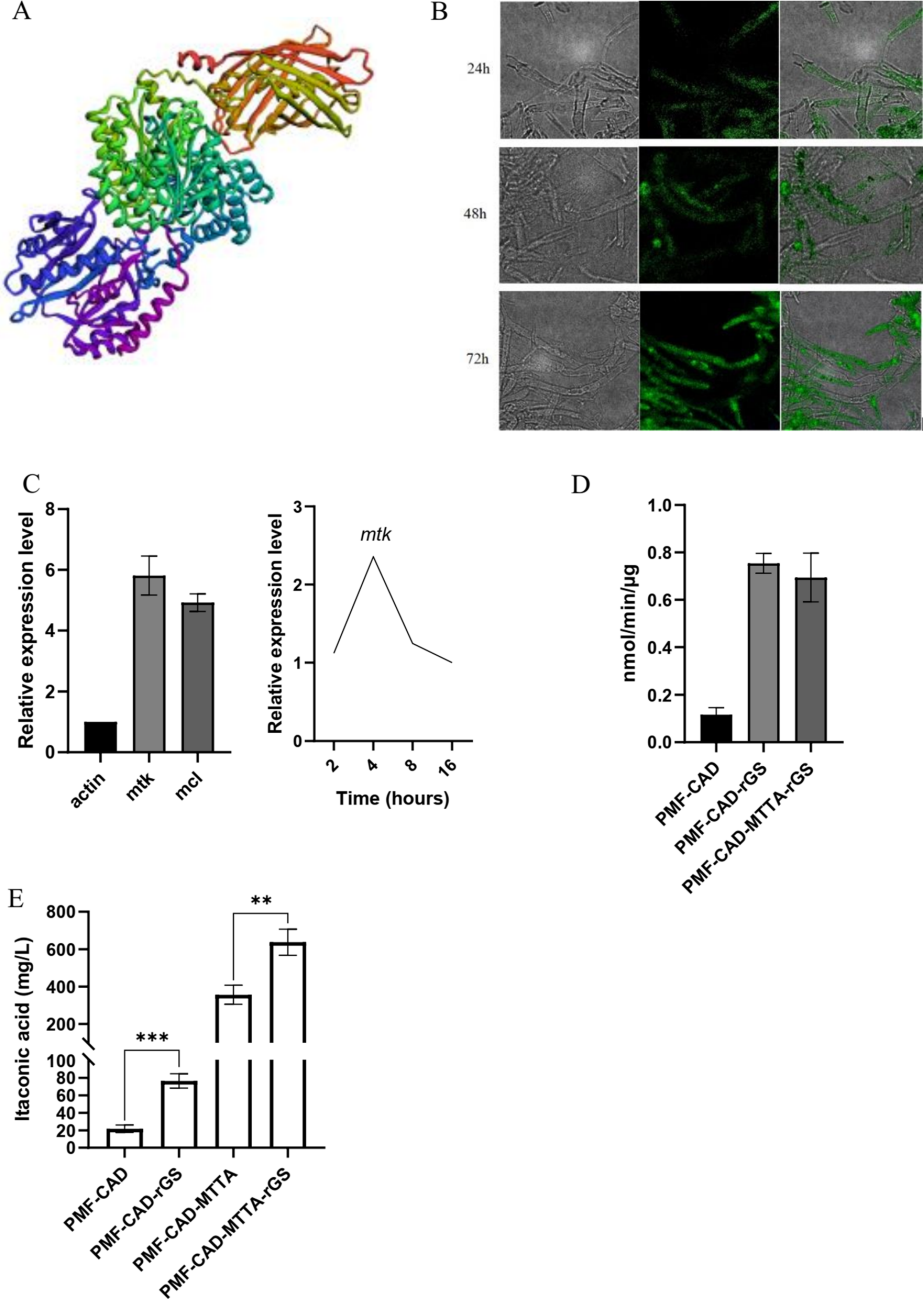
**A** MTK protein structure prediction. The structure of the designed protein (including SucC, SucD, Linker and GFP) was predicted using RoseTTAFold online website (https://robetta.bakerlab.org). **B** Green fluorescence images of the MTK::GFP fusion expressed strain at 24 h, 48 h, 96 h. Left, images obtained under bright field; Middle, images obtained under excitation light; Right, merged images. **C** Expression levels of *mtk* and *mcl* relative to *actin* after 4 h induction by Avicel (left); Expression levels of *mtk* at 2, 4, 8 h of induction with Avicel relative to 16 h of induction (right). **D** MTK–MCL conjugation enzyme activity. **E** Itaconic acid concentrations of different recombinant strains. *T* tests were conducted to evaluate statistical significance at *p* < 0.01 (**) and *p* < 0.001 (***)

The first step of the reverse glyoxylate shunt is to generate Malyl-CoA from malic acid, which can be completed by ATP-dependent malate thiokinase (MTK), of which MTK is derived from *Methylococcus capsulatus* [37]. MTK consists of two sequences, sucC and sucD. Due to the complexity of the enzyme, we first verified whether it can be expressed in *N. crassa*. A linker with three repeated GGSG sequences was designed to fusion SucC and SucD in *N. crassa* [38]. The protein sequence of the designed target gene was used to predict the protein structure of the target gene using RoseTTAFold online website, including SucC subunit (blue–purple), Suc D subunit (green) and GFP (orange–yellow), which was cylindrical bucket-like structure (Fig. 4A). According to the predicted results, it can be preliminarily concluded that the binding of SucC and SucD with the previously selected Linker protein did not affect the effective folding of the amino acid sequence of the two subunits. The mycelia of PMF–MTK strain cultured for 24 h, 48 h and 72 h were prepared and the fluorescence signal was observed under the excitation of blue light source (488 nm) (Fig. 4B). Therefore, MTK derived from* M. capsulatus* could be expressed in *N. crassa*.

In the second step, Malyl-CoA is further cleaved to acetyl-CoA and glyoxylic acid, which is completed by Malyl-CoA lyase (MCL) derived from *Rhodobacter sphaeroides* [39]. MCL was inserted into PMF272–CAD plasmid in an independent expression cassette to obtain strain PMF–CAD–MCL. At the same time, strain PMF–CAD–rGS was obtained by inserting MTK expression cassette after MCL expression cassette. RT-PCR analysis showed that the expression of MTK and MCL in PMF–CAD–MTTA–rGS strain was 5.8-fold and 4.9-fold higher than that of *N. crassa actin*, respectively. In particular, the expression of *mtk* reached its highest level at 4 h (Fig. 4C). The activities of MTK and MCL were detected by MTK–MCL coupled enzyme activity assay (Fig. 4D). The results showed that there was significant activity in the cell lysate. This proves that the rGS can be constructed in *N. crassa* by using the MTK and MCL. Through fermentation verification, the titer of strain PMF–CAD–rGS was 76.5 mg/L, which was 3.5 times that of PMF–CAD (Fig. 4E), indicating that rGS could effectively improve the yield of itaconic acid. On the other hand, the concentration of TCA reducing-end metabolites succinic acid, malic acid and fumaric acid in the strain PMF–CAD–rGS decreased on the 4th day of fermentation, while the concentration of oxidizing-end metabolites cis-aconitic acid, 2-oxoeglutaric acid and itaconic acid increased (Fig. 3A), suggesting that the increase of itaconic acid production in fermentation broth was indeed due to the construction of rGS.

To further increase the content of the precursor cis-aconite acid in the cytoplasm, the cis-aconite transporter MTTA from *Aspergillus terreus* was expressed on the basis of constructing the rGS. Our previous studies have shown that MTTA expression could significantly increase itaconic acid production [12]. Figure 4E shows that the titer of strain PMF–CAD–MTTA was 356.4 mg/L, and the highest titer of PMF–CAD–MTTA–rGS was 637.2 mg/L, indicating that the rGS could further enhance the itaconic acid titer on the basis of the expression of MTTA. Through the above optimization, more carbon source was pulled to itaconic acid synthesis, and its titer was greatly improved compared with the initial strain PMF–CAD.

### Co-optimization of cellulase and metabolism promotes itaconic acid synthesis

**Fig. 5:**
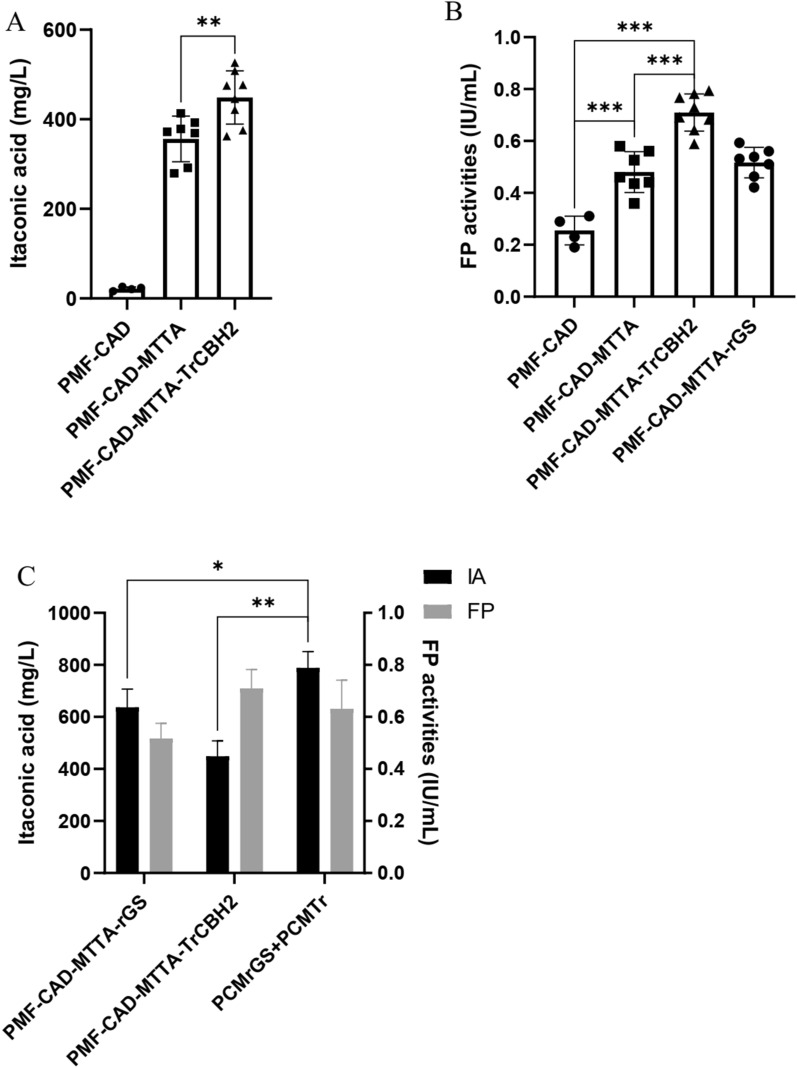
**A** Itaconic acid concentrations of the strain PMF–CAD, PMF–CAD–MTTA and PMF–CAD–MTTA–TrCBH2. **B** FP activities of the strain PMF–CAD, PMF–CAD–MTTA, PMF–CAD–MTTA–TrCBH2 and PMF–CAD–MTTA–rGS. **C** Itaconic acid concentrations and FP activities of the strain PMF–CAD–MTTA–rGS, PMF–CAD–MTTA–TrCBH2 and their mixed culture (PCMrGS + PCMTr). *T* tests were conducted to evaluate statistical significance at t *p* < 0.05 (*), *p* < 0.01 (**) and *p* < 0.001 (***)

In view of the obvious effect of MTTA on itaconic acid synthesis in *N. crassa*, it was co-expressed with TrCBH2 to study the synthesis of itaconic acid from cellulose. The CAD, MTTA and TrCBH2 genes were expressed in tandem in *N. crassa*. As shown in Fig. 5A, the strain PMF–CAD–MTTA–TrCBH2 promoted the titer of itaconic acid, and the titer was 448.7 mg/L. The expression of TrCBH2 also resulted in a significant increase in FP activity compared with PMF–CAD–MTTA (Fig. 5B). Although PMF–CAD–MTTA did not optimize cellulase components, its FP activity was significantly higher than that of PMF–CAD. Our previous research has reported and analyzed this phenomenon in detail. The synthesis of itaconic acid weakened the TCA cycle and electron transport chain, which reduced the productivity of the entire system. Under the condition of low energy, the cellulase system was adjusted accordingly, which increased the synthesis of LPMOs related enzymes, and thus improved the overall cellulase activity [12]. Strain PMF–CAD–MTTA–TrCBH2 was constructed to further expressed the cellobiohydrolase 2 in the strain PMF–CAD–MTTA to improve the overall cellulase activity, thereby increasing the concentration of itaconic acid. In contrast, the FP activity of PMF–CAD–MTTA–rGS was not significantly improved compared with PMF–CAD–MTTA, which may be because itaconic acid synthesis needs to reach a certain threshold to cause significant changes in cellulase system. For example, the itaconic acid production of PMF–CAD–MttA was 18 times higher than that of PMF–CAD. Although rGS could further increase itaconic acid production, the increase was not enough to cause the change of cellulase system. At the same time, to further investigate the interaction between cellulose degradation and itaconic acid synthesis, the optimal strain PMF–CAD–MTTA–rGS (PCMrGS) for itaconic acid synthesis and the optimal strain PMF–CAD–MTTA–TrCBH2 (PCMTr) for FP activity were mixed in the same system. Using Avicel as substrate, the two strains were simultaneously inoculated in equal proportions, and the itaconic acid titer and FP activity after 72 h fermentation are shown in Fig. 5C. As expected, the mixed strains produced more itaconic acid than the single strains, indicating a synergistic effect between them. However, there was little change in cellulase activity.

### Synthesis of itaconic acid from lignocellulose

**Fig. 6:**
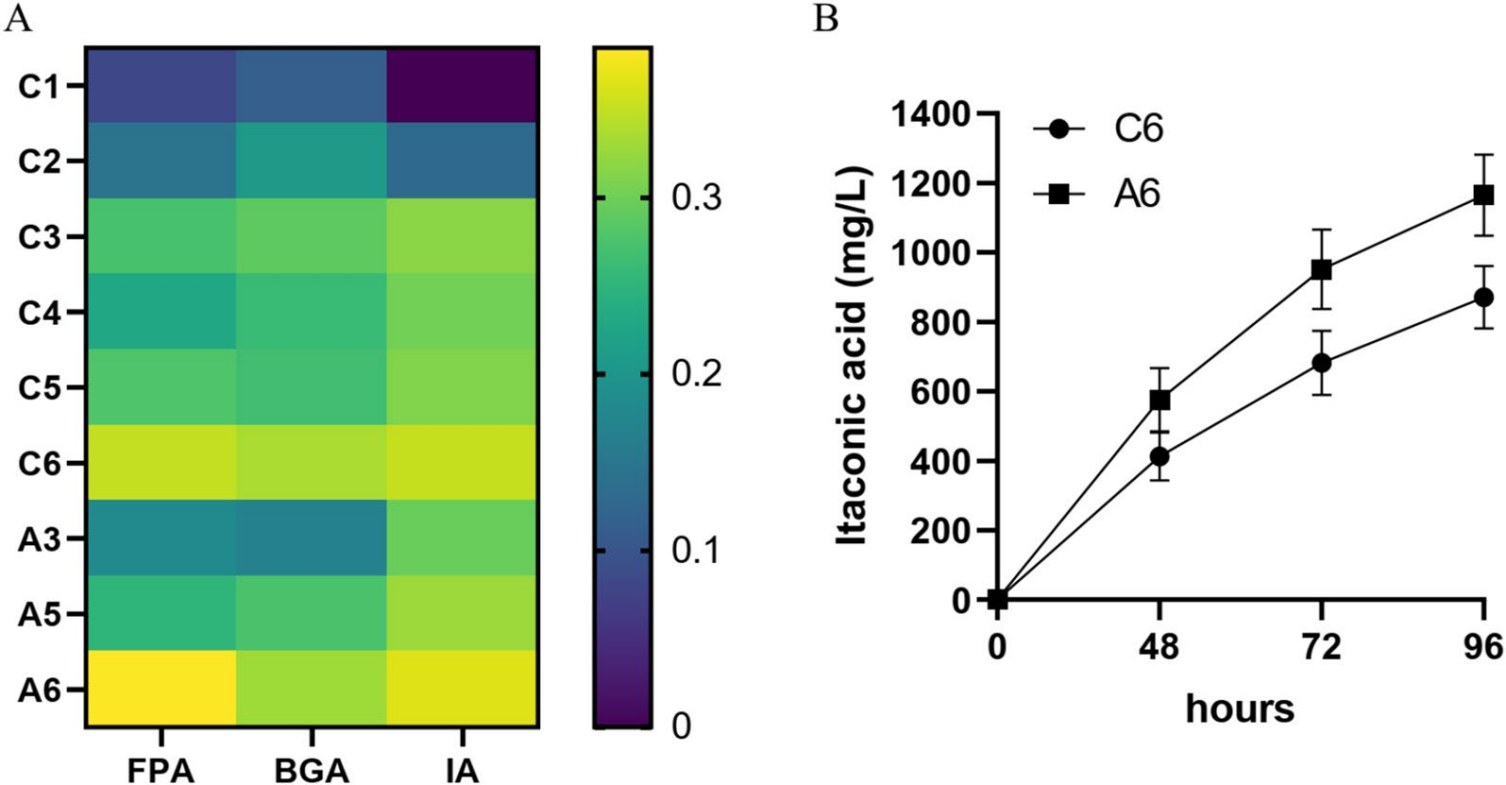
**A** Effects of different medium components on FP activities (FPA), β-glucosidase activities (BGA) and itaconic acid concentrations (IA). The colors were according to the averages of lg values of the activity or concentration of each sample. C1–only 0.5 g corn stover was added; C2–0.5 g corn stover with inorganic salt mixture; C3–C2 added 0.025 g ammonium sulfate; C4–C2 added 0.25 g ammonium sulfate; C5–C3 added trace elements mixture; C6–C3 added 0.3% talcum powder; A3–0.5 g Avicel with inorganic salts mixture and 0.025 g ammonium sulfate; A5–A3 added trace element mixture; A6–A5 added 0.3% talcum powder. **B** Concentrations of itaconic acid at 48, 72 and 96 h using C6 or A6 medium

In this section, corn stover was used as a substrate for the synthesis of itaconic acid. Since the Vogel’s classical medium used by *N. crassa* for theoretical research has been used in the previous experiments, which is complicated to prepare and expensive, this section simplified the medium for corn stover substrate. The preparation of medium is shown in the section of Materials and methods. The activities of filter paper (FPA) and β-glucosidase (BGA) were very low in the medium with water (C1) and inorganic salt (C2), and there was no itaconic acid synthesis (Fig. 6A). The addition of nitrogen sources has an obvious positive effect on the production of cellulase. Natural corn stover generally contains 1.07% dry matter nitrogen [40], which is not enough for the massive secretion of cellulase. In this experiment, the addition of 0.5 g/L (C3) or 5 g/L (C4) ammonium sulfate could significantly improve the cellulase activity, but 0.5 g/L ammonium sulfate was better. When corn stover was used as substrate, the addition of trace element (C5) had little effect on cellulase activity and itaconic acid titer. However, when Avicel was used as substrate, the effect of adding trace elements (A5) was better than that of not adding (A3). Different from Avicel, the components of corn stover are more complex and naturally contain a variety of mineral elements such as P, K, Na, Ca, Mg, Fe, Cu and Zn [40]. In this experiment, the effect of adding Fe, Zn, Mn and Co to the Avicel medium (A5) was not different from that of the natural substrate (C3). Therefore, C3 and A5 was the best medium for the substrate of corn stover and Avicel, respectively.

The mycelium of *N. crassa* is long, and it is easy to wrap and stick to the wall when it is liquid culture in shaking flask. Oxygen and nutrients in the medium cannot be effectively transferred to the mycelium wrapped around the interior, which is easy to cause slow metabolism or death of this part of the mycelium. The main component of talcum powder is water-containing magnesium silicate, which has good lubricity. Our previous study found that adding 0.3% talcum powder with particle size less than 350 mesh during fermentation could effectively prevent mycelium entanglement, and make it loose and more conducive to cellulase secretion. Talcum powder helped to enhance the itaconic acid concentration of PCMrGS + PCMTr (the strains PCMrGS and PCMTr inoculated in equal proportions) to 1165.1 mg/L with Avicel as the substrate (A6), which was 47.7% higher than that of the Vogel’s medium (Fig. 6B). Meanwhile, the titer reached 871.3 mg/L when corn stover was used as substrate (C6). This may be because after adding talcum powder, filamentous microorganisms grew on the surface of these microparticles and formed relatively loose mycelium pellets, which made the transfer of nutrients and oxygen inside the pellet easier and more thorough [41].

## Discussion

In this study, we optimized and analyzed the process of cellulose conversion to itaconic acid in *N. crassa*, which involved the expression of several heterologous genes. The regulation of gene expression level is a prerequisite for rational strain optimization through metabolic engineering. In both model filamentous fungi and industrially important fungi, a wide variety of constitutive or inducible promoters involved in host cell physiological processes have been evaluated for applications. In contrast to *Trichoderma* and *Aspergillus*, few studies have specifically evaluated *N. crassa* promoters expressing heterologous genes. In the study on *T. reesei*, the promoter of the cellobiohydrolase CBH-1 was often used to express extracellular cellulase [42]. In this study, the promoter of cellobiohydrolase CBH-1 of *N. crassa* could also be used for cellobiohydrolase expression, and this promoter was more effective in the expression of TrCBH2 derived from *T. reesei* compared with *Pccg-1*. More interestingly, this promoter could also be used to express the intracellular protein MTTA (Fig. 5 A). TEF-1 is a translation extension factor, and transcriptome data indicate that the protein can be expressed in large quantities on the medium with glucose, xylose or arabinose as single carbon source [43]. Its transcription level is similar to that of glyceraldehyde 3-phosphate dehydrogenase under glucose induction, both of which can be expressed in large quantities, so they are relatively ideal for expressing constitutive promoters of heterologous genes. However, when Avicel was used as substrate, the rate of monosaccharide production was low, so the effect of *Ptef-1* and *Pgpd* was less than that of inducible promoters *Pccg-1* and *Peas*. The clock-related genes and cellulase related genes mentioned above are highly expressed in a variety of biomass and starvation environments [36]. Therefore, the gene expression strategy consisting of promoters of these genes established in this study is not only suitable for Avicel, but also for more complex substrates such as corn stover (Fig. 6).

Several important cellulase components were overexpressed in the itaconic acid synthetic strain of *N. crassa*. It can be seen that increasing the CBH2 of *N. crassa* was more effective in improving the degradation efficiency of cellulose. Cellulase cellobiohydrolase components CBH1 and CBH2 differ in that they cut cellulose molecules from the non-reducing and reducing ends, respectively, to produce cellobiose [44]. In *T. reesei*, the activity of CBH2 is higher than that of CBH1, but the content is lower. Overexpression of CBH2 can significantly improve the total cellulase activity [45]. In *N. crassa*, CBH1 is the most highly expressed cellulase component, accounting for 39.5% of the total protein, while CBH2 (GH6-2) accounts for only 13.4% [46]. Considering that CBH1 and CBH2 components are difficult to obtain separately in *N. crassa*, we expressed these two components in *Pichia pastoris,* respectively, and purified them to obtain CBH1 and CBH2. The purified CBH1 and CBH2 were added to the cellulase of *N. crassa,* respectively, and the enzymatic hydrolysise experiment showed that CBH2 was more helpful to improve the yield [47]. Therefore, the CBH2 of *N. crassa* is the key factor to enhance the overall cellulase activity. The CBH2 expression derived from *T. reesei* was better and more stable than that of *N. crassa* (Fig. 2B). This is because when homologous genes exist in the chromosomes of *N. crassa*, gene silencing at the transcriptomic level may occur in the vegetative growth phase of the strain, and the probability of silencing is about 30% [48]. In this study, cellulase activity of some strain expressing GH6-2 did not change, or even decreased. Therefore, it is generally necessary to screen multiple transformants and carry out multiple rounds of fermentation experiments to verify the overexpression of genes. There was a significant positive correlation between itaconic acid production and cellulase activity (Fig. 2G), but in the same strain, it was limited to improve itaconic acid production by optimizing cellulase, and the key was to improve carbon source conversion through metabolic regulation.

Through the forward and reverse optimization of TCA cycle, the precursor cis-aconitoic acid could be increased, so as to increase the production of itaconic acid. The rGS was established to convert malic acid and succinic acid into isocitric acid, from which itaconic acid was synthesized. The key enzymes of the rGS, MTK and MCL, were derived from bacteria, and their availability was verified by the detection of green fluorescent protein and MTK–MCL conjugation enzyme activity (Fig. 4). The itaconic acid titer of the strain PMF–CAD–rGS was significantly improved by fermentation verification. MTTA is a mitochondrial tricarboxylate transporter, the best substrate of which is cis-aconitic acid [49]. This protein plays an important role in the synthesis of itaconic acid from yeast [50]. Our study showed that MTTA not only significantly increased itaconic acid production in improving the synthesis of *N. crassa*, but also caused metabolic changes that were sufficient to affect cellulase synthesis. MTTA is able to transport cis-aconitic acid accumulated in mitochondria to the cytoplasm, thereby introducing more carbon source from the oxidative branch of the TCA cycle into the itaconic acid synthesis pathway, resulting in the inflow of carbon source at the reducing end of the TCA cycle is reduced after the expression of MTTA [12]. In the TCA cycle, the conversion of 2-oxoglutaric acid to succinyl-CoA is irreversible, so the TCA cycle is irreversible in nature [51]. The establishment of rGS is equivalent to reversing the TCA cycle and establishing a fast channel for the conversion of the metabolites in reductive branch of the TCA cycle into oxidative branch. Then, we further constructed the rGS enhanced the inflow of carbon sources such as malic acid, fumaric acid and succinic acid to itaconic acid. Although the increase of itaconic acid concentration was not as significant as that of PMF–CAD–rGS (3.5 times), it was still significantly higher than that of PMF–CAD–MTTA. This study could not continue to optimize isocitrate lyase (ICL) due to limited screening marker. The ICL of *N. crassa* is easily inhibited by intermediate metabolites of glycolysis or TCA cycle [52], which greatly limits the better role of rGS. In addition, if the rGS is built directly into mitochondria, it may greatly improve its efficiency. This is because higher local enzyme concentrations can be achieved in mitochondria, and it also eliminates the need to export rGS intermediate metabolites to mitochondria, but on the other hand it will increase the burden of cis-aconitic acid transport by MTTA.

The simultaneous expression of TRCBH2 and MTTA can be regarded as a “push” and “pull” coordination between them. By expressing TrCBH2, cellulase activity was enhanced, more fermentable sugars were produced, and carbon sources were “pushed” to cell metabolism. At the same time, the expression of MTTA “pulled” the carbon source in the cell metabolism process to the itaconic acid synthesis pathway, and the two cooperated to increase the production of itaconic acid (Fig. 5A). In this process, the co-expression of MTTA and TrCBH2 had a better effect on the promotion of FP activity than the expression of TrCBH2 alone, indicating that the optimization of metabolic pathway should also take into account the influence on cellulase synthesis during the optimization of CBP strains. The metabolic optimization through the expression of MTTA and the construction of rGS was more efficient than that of the cellulase optimization, but it was undeniable that the co-optimization of the two could more effectively improve itaconic acid production (Fig. 5A, C). Since *N. crassa* is not a natural itaconic acid producing strain, the metabolic pathway needs to be further optimized. At present, the optimization efficiency of cellulase is relatively low. However, through metabolic engineering optimization, more carbon source flew into the itaconic acid synthesis pathway, and the optimization of cellulase became also important. The “push and pull” strategy has achieved good results in the process of CBP using microbial consortium. For example, the use of *T. reesei*–*Saccharomyces cerevisiae* to degrade lignocellulose to synthesize gluconic acid or single cell protein [53]. The modification of *T. reesei* was to synthesize cellulase in large quantities, while the modification of *S. cerevisiae* was needed to optimize the metabolic pathway as much as possible. Different from this method, it is more difficult to optimize CBP by using a single microorganism, and it is necessary to consider the coordination of cellulase production and product synthesis at the same time, and establish a minimized cell transformation system. This study attempted to simultaneously optimize cellulase synthesis and itaconic acid synthesis, which initially formed a “push and pull” of carbon source in the same microbial system and improved the itaconic acid production (Fig. 5). Subsequent studies will continue to carry out more gene integration and optimization on this basis. Using a single microorganism for CBP can reduce the inconvenience caused by multi-strain culture in industrial production and the complexity of regulation between different strains [54–56], which is a simpler mode of industrial production. Although the current titer is still not high, with the development of synthetic biology, the simulation of cell minimization production, and precise cellular regulation, the titer is expected to be greatly increased.

## Conclusion

A CBP system for the conversion of cellulose to itaconic acid by the filamentous fungus *N. crassa* was established and optimized. The promoters from *N. crassa* used to express heterologous genes were compared. Among the different cellulase components, CBH2 was identified as the main cellulase that affected cellulase activity and itaconic acid production. The itaconic acid precursor cis-aconitic acid was accumulated and itaconic acid production was increased by establishing the reverse glyoxylate shunt and expressing cis-aconite transporter MTTA. Simultaneous optimization of cellulase and metabolic pathways significantly increased itaconic acid production, and the final titers of itaconic acid were 1165.1 mg/L and 871.3 mg/L, respectively, when Avicel and corn stover were used as the sole carbon source. This study confirmed the potential applications of filamentous fungi for direct conversion of lignocellulose to itaconic acid.

### Supplementary Information

**Additional file 1: Figure S1.** Main plasmids used in this study. (A) Plasmids pMF-272-Pccg1/Peas/Pcbh1/Pgh6-2/Pgh11-2/Ptef1/Pgpd/Ppda-CAD were used to compare the expression of CAD in *N. crassa*. (B) Plasmids pMF-272-Pccg1-CBH1/GH6-2/GH5-1/GH3-4/AsBGA/TrCBH2 were used to compare the effects of different cellulases. (C) Plasmids pMF-272-Pccg1-CAD-Pcbh1-CBH1/GH6-2/GH5-1/GH3-4/AsBGA/TrCBH2 were used to compare the effects of different cellulase and CAD co-expression. (D) Plasmid pMF-272-Pccg1-MTK was used to verify the expression of MTK in *N. crassa*. The plasmids pUC19-MTK-HPH (F) and pMF-272-Pccg1-CAD-Pes-MCL (E) or pMF-272-Pccg1-CAD-Pcbh1-MTTA-Pes-MCL (G) were used to construct *N. crassa* PMF-CAD-rGS or *N. crassa* PMF-CAD-MTTA-rGS. (H) Plasmid pMF-272-Pccg1-CAD-Pcbh1-MTTA-Pcbh1-TrCBH2 was used to construct *N. crassa* PMF-CAD-MTTA-TrCBH2. **Figure S2.** PCR amplified the promoter sequence. **Figure S3.** Strain construction process using Pcbh1 as the CAD promoter. (A) Pcbh1 promoter sequence was amplified by PCR. M:Trans2K Plus DNA Marker, 1–6:Pcbh1 (B) PCR identification of vector Blunt-Pcbh1. 1–22: Blunt-Pcbh1 (C) Identification of recombinant plasmid pMF272-CAD. 1–6: pMF272-CAD. (D) Double enzyme digestion of pMF272-CAD recombinant plasmid. (E) Cloning vector Blunt-Pcbh1 double enzyme digestion. (F) Colony PCR identification of recombinant plasmid pMF-CAD-Pcbh1. **Figure S4.** Construction of cellulase overexpression strain. (A) PCR amplification of Pcbh-1 promoter sequence (1 and 2), gh3-4 sequence (4), and cbh1 sequence (B, 1 and 2). (C) Identification of expression vector containing cbh1 gene. (D) PCR screening of gh3-4 gene expression vectors. (E) Genome PCR for vector transformation screening 1,2,3: cbh1; 4,5: gh3-4. **Figure S5.** Construction of MTK, MCL expression strain. (A) PCR amplification of MTK (lines 1 ~ 3). (B) Colony PCR for identification of MTK expression cassette (C) PCR amplification of GFP (1) and terminator fragments (2). Identification of expression vector containing cbh1 gene. Genome PCR for MTK expression (D) and MCL expression (E) vector transformation screening. **Figure S6.** Construction of CAD, MTK and MCL co-expression strain. (A) PCR amplification of 5′ fragment (lines 1 ~ 3). (B) PCR amplification of 3′ fragment (lines 1 ~ 3) and hph fragment (lines 4 and 5). (C) PCR amplification of MTK cassette. (D) Identification of expression vector containing 5′ fragment and hph fragment. **Table S1.** Plasmids used in this study. **Table S2.** Strains used in this study. **Table S3.** Primer list of CAD expression and promoter optimization. **Table S4.** Primer list of cellulase expression. **Table S5.** Primer list of CAD and cellulase co-expression. **Table S6**. Primer list of MTK, MCL and MTTA expression. **Table S7.** RT-PCR Primers.

## Data Availability

All data are included in this published article and its additional information files.
