# Supplementary material for: Improved consolidated bioprocessing for itaconic acid production by simultaneous optimization of cellulase and metabolic pathway of Neurospora crassa
Source: Biotechnol Biofuels Bioprod. 2024 Apr 29;17:57. doi: 10.1186/s13068-024-02505-5 (PMC11059683; doi:10.1186/s13068-024-02505-5)
Supplement: Supplementary file 1 — Additional file 1: Figure S1. Main plasmids used in this study. (A) Plasmids pMF-272-Pccg1/Peas/Pcbh1/Pgh6-2/Pgh11-2/Ptef1/Pgpd/Ppda-CAD were used to compare the expression of CAD in N. crassa. (B) Plasmids pMF-272-Pccg1-CBH1/GH6-2/GH5-1/GH3-4/AsBGA/TrCBH2 were used to compare the effects of different cellulases. (C) Plasmids pMF-272-Pccg1-CAD-Pcbh1-CBH1/GH6-2/GH5-1/GH3-4/AsBGA/TrCBH2 were used to compare the effects of different cellulase and CAD co-expression. (D) Plasmid pMF-272-Pccg1-MTK was used to verify the expression of MTK in N. crassa. The plasmids pUC19-MTK-HPH (F) and pMF-272-Pccg1-CAD-Pes-MCL (E) or pMF-272-Pccg1-CAD-Pcbh1-MTTA-Pes-MCL (G) were used to construct N. crassa PMF-CAD-rGS or N. crassa PMF-CAD-MTTA-rGS. (H) Plasmid pMF-272-Pccg1-CAD-Pcbh1-MTTA-Pcbh1-TrCBH2 was used to construct N. crassa PMF-CAD-MTTA-TrCBH2. Figure S2. PCR amplified the promoter sequence. Figure S3. Strain construction process using Pcbh1 as the CAD promoter. (A) Pcbh1 promoter sequence was amplified by PCR. M:Trans2K Plus DNA Marker, 1–6:Pcbh1 (B) PCR identification of vector Blunt-Pcbh1. 1–22: Blunt-Pcbh1 (C) Identification of recombinant plasmid pMF272-CAD. 1–6: pMF272-CAD. (D) Double enzyme digestion of pMF272-CAD recombinant plasmid. (E) Cloning vector Blunt-Pcbh1 double enzyme digestion. (F) Colony PCR identification of recombinant plasmid pMF-CAD-Pcbh1. Figure S4. Construction of cellulase overexpression strain. (A) PCR amplification of Pcbh-1 promoter sequence (1 and 2), gh3-4 sequence (4), and cbh1 sequence (B, 1 and 2). (C) Identification of expression vector containing cbh1 gene. (D) PCR screening of gh3-4 gene expression vectors. (E) Genome PCR for vector transformation screening 1,2,3: cbh1; 4,5: gh3-4. Figure S5. Construction of MTK, MCL expression strain. (A) PCR amplification of MTK (lines 1 ~ 3). (B) Colony PCR for identification of MTK expression cassette (C) PCR amplification of GFP (1) and terminator fragments (2). Identification of expression vector conta [file 13068_2024_2505_MOESM1_ESM.docx]

**Supplementary material for Publication**

Improved consolidated bioprocessing for itaconic acid production by simultaneous optimization of cellulase and metabolic pathway of *Neurospora crassa*

Chen Zhao ^1,2,^*, Jiajia Zhao ^1,3^, Jingjing Han ^1,2^, Yaojie Mei ^1,2^, Hao Fang ^4,5,^*

^1^ College of Life Sciences, Northwest A&F University, Yangling 712100, Shaanxi, China.

^2^ Biomass Energy Center for Arid and Semi-arid Lands, Northwest A&F University, Yangling 712100, Shaanxi, China.

^3^ The Second Department of Vaccine, Lanzhou Institute of Biological Products Co., Ltd., Lanzhou 730046, China.

^4^ Key Laboratory of Biomass Chemical Engineering of Ministry of Education, College of Chemical and Biological Engineering, Zhejiang University, Hangzhou 310027, China.

^5^ ZJU-Hangzhou Global Scientific and Technological Innovation Center, Zhejiang University, Hangzhou 311215, Zhejiang, China.

1.The main plasmids used in this study.

B

A

D

C

H

G

F

E

**Fig.S1** The main plasmids used in this study.

(A) The plasmids pMF-272-Pccg1/Peas/Pcbh1/Pgh6-2/Pgh11-2/Ptef1/Pgpd/Ppda-CAD were used to compare the expression of CAD in *N. crassa*. (B) The plasmids pMF-272-Pccg1-CBH1/GH6-2/GH5-1/GH3-4/AsBGA/TrCBH2 were used to compare the effects of different cellulases. (C) The plasmids pMF-272-Pccg1-CAD-Pcbh1-CBH1/GH6-2/GH5-1/GH3-4/AsBGA/TrCBH2 were used to compare the effects of different cellulase and CAD co-expression. (D) The plasmid pMF-272-Pccg1-MTK was used to verify the expression of MTK in *N. crassa*. The plasmids pUC19-MTK-HPH (F) and pMF-272-Pccg1-CAD-Pes-MCL (E) or pMF-272-Pccg1-CAD-Pcbh1-MTTA-Pes-MCL (G) were used to construct *N. crassa* PMF-CAD-rGS or *N. crassa* PMF-CAD-MTTA-rGS. (H) The plasmid pMF-272-Pccg1-CAD-Pcbh1-MTTA- Pcbh1-TrCBH2 was used to construct *N. crassa* PMF-CAD-MTTA-TrCBH2.

2. Construction process of the main recombinant strains

The genomic DNA of wild type *N.crassa* (FGSC2489) was extracted as the template, and the promoter sequences at about 1.0 kb upstream of the selected gene were amplified by PCR. The primers used in the experiment are shown in Table S3. The results of promoter amplification are shown in Fig S2.

Pcbh1 was used as an example to introduce the strain construction process in detail.

PCR amplified Pcbh1 sequence: The results were shown in Fig S3A. The band size was about 950bp. Identification of *E. coli* cloning vector Blunt-Pcbh1: 22 single colonies were selected from the cloning vector for colony PCR identification. The detection results were shown in S3B, among which 18 samples showed target bands with a size of about 950bp, which met the requirements of the experimental design. Samples No. 1, 2, 7, and 8 were selected for further identification and sequencing. The correctness of the recombinant plasmid pMF272-CAD was verified: the recombinant plasmid was transformed, single colony was selected for culture, and the plasmid was extracted. Cis-aconite decarboxylase cadF and cadR were used as primers (Table S3), and the recombinant plasmid was identified by PCR. The PCR electrophoresis results are shown in Fig. S3C. The band size detected by electrophoresis of the recombinant plasmid is about 1.5kb, which was basically consistent with the reference, that is, the gene size of cis-aconite decarboxylase was 1487bp, and the vector structure was correct. Double enzyme digestion of recombinant plasmid and promoter fragment: Restriction enzymes XbaI and NotI were used to double enzyme digestion of pMF272-CAD plasmid and cloned vector. a. Enzyme digestion of the skeleton carrier: the size of pMF272-CAD plasmid is 9935bp, and the enzyme digestion electrophoresis test is shown in Fig. S3D, with two bands of 9kb and 1kb, indicating good enzyme digestion effect. The 9k size band was subsequently cut and recovered to be used as the skeleton carrier of the recombinant plasmid. b. Double enzyme digestion of the cloning vector: The promoter cloning vector was cut by restriction enzymes NotI and XbaI respectively, and the glue was recovered. The detection was shown in Fig. S3E. There were two bands of 4kb and 1kb. Identification of recombinant plasmid pMF272-CAD-Pcbh1:24 single colonies were selected from recombinant plasmid pMF272-CAD-Pcbh1 for colony PCR identification. The detection results were shown in Fig. S3F, among which 24 samples showed target bands with a size of about 950bp, which met the requirements of experimental design. Samples 1, 2 and 7 were selected for further identification and sequencing.

**Fig.S2** PCR amplified the promoter sequence

**Fig.S3** Strain construction process using Pcbh1 as the CAD promoter

(A) Pcbh1 promoter sequence was amplified by PCR. M:Trans2K Plus DNA Marker,1-6:Pcbh1(B) PCR identification of vector Blunt-Pcbh1. 1-22：Blunt-Pcbh1 (C) Identification of recombinant plasmid pMF272-CAD. 1-6：pMF272-CAD. (D) Double enzyme digestion of pMF272-CAD recombinant plasmid. (E) Cloning vector Blunt-Pcbh1 double enzyme digestion. (F) Colony PCR identification of recombinant plasmid pMF-CAD-Pcbh1.

Construction of cellulase overexpression/heterologous expression strains (Take CBH1 and GH3-4 for example). For overexpression, using wild type *N. crassa* genome as template, Pcbh-1, gh3-4 and cbh1 sequences were amplified by PCR, as shown in Fig.S4AB. The primers are shown in Table S4. The bands of 900bp, 2400bp and 2800bp were bright in color. The pMF272-gh3-4 and pMF272-cbh1 were constructed and transferred into *E. coli*. After the transformation of pMF272-cbh-1 recombinant plasmid, 20 single colonies were selected for colony PCR identification, among which 2 samples showed target bands with a size of about 2800bp. The plasmid PCR results are shown in Fig.S4C. Similarly, for gh3-4, 24 single colonies were selected for colony PCR identification, and the detection results were shown in Fig.S4D, among which 1 sample showed a target band with a size of about 3000bp (including promoter). the screening results of strains expressing CBH1 and GH3-4 are shown in Fig.S4E.

**Fig.S4** Construction of cellulase overexpression strain

(A) PCR amplification of Pcbh-1 promoter sequence (1 and 2), gh3-4 sequence (4), and cbh1 sequence (B, 1 and 2). (C) Identification of expression vector containing cbh1 gene. (D) PCR screening of gh3-4 gene expression vectors. (E) Genome PCR for vector transformation screening 1,2,3: cbh1; 4,5: gh3-4

The total length of MTK gene was 2100bp, including two subunits SucC and SucD and a linker sequence. Fig.S5A shows the results of MTK and GFP (717bp). Primers are shown in Table S6. The trpC terminator of *Aspergillus nidulatus* were amplified by PCR. Results as shown in Fig.S5C, the length of trpC fragment was 712bp. MTK gene fragments, GFP, and terminator fragments were linked to the vector, and 20 single colonies were selected for colony PCR identification after transformation. Target gene bands appeared in colonies No. 3 and No. 21, and the detected fragments included the total length of MTK-GFP-trpC 3526bp (Fig.S5B). After the transformation of *N. crassa*, 6 strains were selected for verification. As shown in Fig.S5D, the results of MTK-GFP-trpC gene fragment (3526bp) upstream and downstream primers were used to verify the results of strain 1-6. Strains 1-6 were verified by PCR using upstream and downstream primers of GFP gene fragment (717bp) for strains 7-12. Similarly, the screening results of strains expressing MCL (980bp) are shown in Fig.S5E.

**Fig.S5** Construction of MTK, MCL expression strain

(A) PCR amplification of MTK (lanes 1~3). (B) Colony PCR for identification of MTK expression cassette (C) PCR amplification of GFP (1) and terminator fragments (2). Identification of expression vector containing cbh1 gene. Genome PCR for MTK expression (D) and MCL expression (E) vector transformation screening.

For construction of strains *N. crassa* PMF-CAD-rGS and *N. crassa* PMF-CAD-MTTA-rGS, CAD, MTTA, MCL were expressed in pMF272. MTK and HPH were connected with plasmid PUC19 and inserted into the downstream site of MCL expression in *N. crassa* by homologous arms. According to the sequence information of MF272 plasmid, the downstream genes of EcoR I were amplified by primers 5flank F/R and 3flank F/R. As shown in Fig.S6A, bands 1-3 are 5 'segment of the upstream homologous arm, with a size of 1550bp. As shown in Fig.S6B, bands 1-3 are 3 'homologous arm with a length of 1510bp, and bands 4 and 5 are hph fragments with a size of 1393bp. The bands 1 and 2 in Fig. S6C are MTK cassette. The plasmid PUC19-MTK-HPH was obtained after the above fragment was connected with PUC19 (kpn I), and the plasmid was transferred into the *N. crassa* recombinant strain. Six transformants were obtained. The sizes of the 5 'fragment and hph fragment of the upstream homologous arm were verified to be 2869bp (Fig. S6D).

**Fig.S6** Construction of CAD, MTK, MCL co-expression strain

(A) PCR amplification of 5 ' fragment (lanes 1~3). (B) PCR amplification of 3 ' fragment (lanes 1~3) and hph fragment (lanes 4 and 5). (C) PCR amplification of MTK cassette. (D) Identification of expression vector containing 5 'fragment and hph fragment.

**Table S1** Plasmids used in this study.

| Plasmids | Descriptions | Source |
| --- | --- | --- |
| pMF-272 | Pccg-1, Ampicillin, his+ | Fungal Genetics Stock Center |
| pUC19 | Lac, Ampicillin | Stored in authors´ laboratory |
| pMF-272-Pccg1-CAD | Recombinant vector carrying *cad1* | This study |
| pMF-272-Peas-CAD | Recombinant vector carrying *cad1* | This study |
| pMF-272-Pcbh1-CAD | Recombinant vector carrying *cad1* | This study |
| pMF-272-Pgh6-2-CAD | Recombinant vector carrying *cad1* | This study |
| pMF-272-Pgh11-2-CAD | Recombinant vector carrying *cad1* | This study |
| pMF-272-Ptef1-CAD | Recombinant vector carrying *cad1* | This study |
| pMF-272-Pgpd-CAD | Recombinant vector carrying *cad1* | This study |
| pMF-272-Ppda-CAD | Recombinant vector carrying *cad1* | This study |
| pMF-272-Pccg1-CAD-Peas-CAD | Recombinant vector carrying *cad1* | This study |
| pMF-272-Pccg1-CBH1 | Recombinant vector carrying *cbh1* | This study |
| pMF-272-Pccg1-GH6-2 | Recombinant vector carrying *gh6-2* | This study |
| pMF-272-Pccg1-GH5-1 | Recombinant vector carrying *gh5-1* | This study |
| pMF-272-Pccg1-AsBGA | Recombinant vector carrying *asbga* | This study |
| pMF-272-Pccg1-TrCBH2 | Recombinant vector carrying *trcbh2* | This study |
| pMF-272-Pccg1-CAD-Pcbh1-CBH1 | Recombinant vector carrying *cad1* and *cbh1* | This study |
| pMF-272-Pccg1-CAD-Pcbh1-GH6-2 | Recombinant vector carrying *cad1* and *gh6-2* | This study |
| pMF-272-Pccg1-CAD-Pcbh1-GH5-1 | Recombinant vector carrying *cad1* and *gh5-1* | This study |
| pMF-272-Pccg1-CAD-Pcbh1-GH3-4 | Recombinant vector carrying *cad1* and *gh3-4* | This study |
| pMF-272-Pccg1-CAD-Pcbh1-AsBGA | Recombinant vector carrying *cad1* and *asbga* | This study |
| pMF-272-Pccg1-CAD-Pcbh1-TrCBH2 | Recombinant vector carrying *cad1* and *trcbh2* | This study |
| pMF-272-Pccg1-MTK | Recombinant vector carrying *mtk* | This study |
| pMF-272-Pccg1-CAD-Pes-MCL | Recombinant vector carrying *cad1* and *mcl* | This study |
| pUC19-MTK-HPH | Recombinant vector carrying *mtk* and *hph* | This study |
| pMF-272-Pccg1-CAD-Pcbh1-MTTA | Recombinant vector carrying *cad1* and *mtta* | This study |
| pMF-272-Pccg1-CAD-Pcbh1-MTTA-Pes-MCL | Recombinant vector carrying *cad1*, *mtta* and *mcl* | This study |
| pMF-272-Pccg1-CAD-Pcbh1-MTTA- Pcbh1-TrCBH2 | Recombinant vector carrying *cad1*, *mtta* and *trcbh2* | This study |

**Table S2** Strains used in this study.

| Strains | Descriptions | Source |
| --- | --- | --- |
| *N. crassa* FGSC 9720 | mus-52::bar his-3 mat A+ | Fungal Genetics Stock Center |
| *N. crassa* FGSC 2489 | Wild type | Fungal Genetics Stock Center |
| *E. coli* DH5α | Used for constructing and amplifying plasmids | Stored in authors´ laboratory |
| PMF | Recombinant strain carrying empty pMF-272 plasmid | This study |
| *N. crassa* Pccg-1(PMF-CAD) | Recombinant strain carrying CAD | This study |
| *N. crassa* Peas | Recombinant strain carrying CAD | This study |
| *N. crassa* Pcbh1 | Recombinant strain carrying CAD | This study |
| *N. crassa* Pgh6-2 | Recombinant strain carrying CAD | This study |
| *N. crassa* Pgh11-2 | Recombinant strain carrying CAD | This study |
| *N. crassa* Ptef1 | Recombinant strain carrying CAD | This study |
| *N. crassa* Pgpd | Recombinant strain carrying CAD | This study |
| *N. crassa* Ppda | Recombinant strain carrying CAD | This study |
| *N. crassa* Pccg-1+ Peas | Recombinant strain carrying CAD | This study |
| *N. crassa* PMF-CBH1 | Recombinant strain carrying CBH1 | This study |
| *N. crassa* PMF-GH6-2 | Recombinant strain carrying GH6-2 | This study |
| *N. crassa* PMF-GH5-1 | Recombinant strain carrying GH5-1 | This study |
| *N. crassa* PMF-AsBGA | Recombinant strain carrying AsBGA | This study |
| *N. crassa* PMF-TrCBH2 | Recombinant strain carrying TrCBH2 | This study |
| *N. crassa* PMF-CAD-CBH1 | Recombinant strain carrying CAD and CBH1 | This study |
| *N. crassa* PMF-CAD-GH6-2 | Recombinant strain carrying CAD and GH6-2 | This study |
| *N. crassa* PMF-CAD-GH5-1 | Recombinant strain carrying CAD and GH5-1 | This study |
| *N. crassa* PMF-CAD-GH3-4 | Recombinant strain carrying CAD and GH3-4 | This study |
| *N. crassa* PMF-CAD-AsBGA | Recombinant strain carrying CAD and AsBGA | This study |
| *N. crassa* PMF-CAD-TrCBH2 | Recombinant strain carrying CAD and TrCBH2 | This study |
| *N. crassa* PMF-CAD-rGS | Recombinant strain carrying CAD, MTK and MCL | This study |
| *N. crassa* PMF-CAD-MTTA | Recombinant strain carrying CAD and MTTA | This study |
| *N. crassa* PMF-CAD-MTTA-rGS | Recombinant strain carrying CAD, MTTA, MTK and MCL | This study |
| *N. crassa* PMF-CAD-MTTA-TrCBH2 | Recombinant strain carrying CAD, MTTA and TrCBH2 | This study |

**Table S3** Primer list of CAD expression and promoter optimization

| Name | Description |
| --- | --- |
| cad1-F | ctcacatcaaccaaatctagaATGACCAAGCAGTCCGCCGACT |
| cad1-R | gataagcttgatatcgaattcTCAGACGAGGGGGCTCTTG |
| Pccg1 F | ctccaccgcggtggaattcTAGAAGGAGCAGTCCATCTGCG |
| Pccg1 R | ggactgcttggtcatgaattcTTTGGTTGATGTGAGGGGTTG |
| Pgh11-2 F | ctccaccgcggtggaattcATATTGCTTGGAGGCTCTAGCCT |
| Pgh11-2 R | ggactgcttggtcatgaattcTTCGATGTCTTTGACCTTAGACTGG |
| Peas F | ctccaccgcggtggaattcACTTGCACTTGGCAACCGAG |
| Peas R | ggactgcttggtcatgaattcTGCTGAAAGAAGCTGGGAGTTG |
| Pcbh1 F | ctccaccgcggtgttaattaaACTTAACGTTACTGAAATCATCAAACAG |
| Pcbh1 R | ggactgcttggtcatttaattaaTCTAGAAAGAAGGATTACCTCTAAACAAG |
| Pgh6-2 F | ctccaccgcggtgttaattaaAGAGACCCGGAAGTCGCCA |
| Pgh6-2 R | ggactgcttggtcatttaattaaTTGTAAGAACTGTTGATGAT |
| Ptef1 F | ctccaccgcggtggaattcACTAGTCTAGAGTGAAGCT |
| Ptef1 R | ggactgcttggtcatgaattcTTTGACGGTTGATGTGCTGA |
| Pgpd F | agggaggcaaacaatgaACTTAACGTTACTGAAATCATCAAACAG |
| Pgpd R | ggtatcgataagcttgatatcgaattcTCTAGAAAGAAGGATTACCTCTAAACAAG |
| Ppda F | cagggaggcaaacaatgaACTTAACGTTACTGAAATCATCAAACAG |
| Ppda R | ggtatcgataagcttgatatcgaattc TCTAGAAAGAAGGATTACCTCTAAACAAG |

**Table S4** Primer list of cellulase expression

| Name | Description |
| --- | --- |
| cbh1 F | cctcacatcaaccaaatctagaATGCTCGCCAAGTTCGCTG |
| cbh1 R | tgatttcagtaacgttaagttctagaTTACACGCACTGGGAGTAATAGTCG |
| gh6-2 F | cctcacatcaaccaaatctagaATGGCTGCCAAGAAGCTCC |
| gh6-2 R | tgatttcagtaacgttaagttctagaTCAGAAAGCGGGGTTAGCG |
| gh5-1 F | cctcacatcaaccaaatctagaATGAAGGCTACGATTCTTGCCA |
| gh5-1 R | tgatttcagtaacgttaagttctagaTTAAGGGGTATAGGTCTTGAGAAGG |
| asbga F | cctcacatcaaccaaatctagaATGCTCGCCAAGTTCGCTG |
| asbga R | tgatttcagtaacgttaagttctagaTCAGTGGACGGTGGGGAGG |
| trcbh2 F | cctcacatcaaccaaatctagaATGCTCGCCAAGTTCGCTG |
| trcbh2 R | tgatttcagtaacgttaagttctagaTCAGAGGAAGGAGGGGTTGG |
| TtrpC F | cctcacatcaaccaaatctagaACTTAACGTTACTGAAATCATCAAACAG |
| TtrpC R | tatcgataagcttgatatcgaattcAAGAAGGATTACCTCTAAACAAGTGTACC |

**Table S5** Primer list of CAD and cellulase co-expression

| Name | Description | |
| --- | --- | --- |
| Pcbh-1 F | | agccccctcgtctgattaattaaCTTGAAGCTGCCAACTCAACC |
| Pcbh-1 R | | gaaggtgcatGGTGAAGATGAGGCTGAACGG |
| gh3-4 F | | catcttcaccATGCACCTTCGAATATTTGCG |
| gh3-4 R | | cccttgctcaccatggaattcATAAACATCAAACTTCCCATTCAACC |
| cbh-1 F | | agccccctcgtctgattaattaaCTTGAAGCTGCCAACTCAACC |
| cbh-1 R | | cccttgctcaccatggaattcCACGCACTGGGAGTAATAGTCG |
| tPcbh-1 F | | agccccctcgtctgattaattaaCTTGAAGCTGCCAACTCAACC |
| tPcbh-1 R | | gccttcatGGTGAAGATGAGGCTGAACGG |
| gh5-1 F | | ctcatcttcaccATGAAGGCTACGATTCTTGCCA |
| gh5-1 R | | cccttgctcaccatggaattcTTAAGGGGTATAGGTCTTGAGAAGG |
| gh6-2 F | | atcttcaccATGGCTGCCAAGAAGCTCC |
| gh6-2 R | | tcagtaacgttaagttctagaTCAGAAAGCGGGGTTAGCG |
| Pcbh1-As F | | agccccctcgtctgattaattaaCTTGAAGCTGCCAACTCAACC |
| Pcbh1-As R | | ttggcgagcatGGTGAAGATGAGGCTGAACGG |
| asbga F | | atcttcaccATGCTCGCCAAGTTCGCTG |
| asbga R | | gataagcttgatatcgaattcTCAGTGGACGGTGGGGAGG |
| Pcbh1-Tr F | | agccccctcgtctgattaattaaCTTGAAGCTGCCAACTCAACC |
| Pcbh1-Tr R | | ttggcgagcatGGTGAAGATGAGGCTGAACGG |
| trcbh2 F | | atcttcaccATGCTCGCCAAGTTCGCTG |
| trcbh2 R | | gataagcttgatatcgaattcTCAGAGGAAGGAGGGGTTGG |

**Table S6** Primer list of MTK, MCL, MTTA expression

| Name | Description |
| --- | --- |
| MTK F | ctcacatcaaccaaatctagaATGAATATCCATGAGTACCAGGCC |
| MTK R | cccttgctcacGAATCTGATTCCGTGTTCCTGC |
| GFP F | atcagattcGTGAGCAAGGGCGAGGAGC |
| GFP R | gtTTACTTGTACAGCTCGTCCATGCC |
| TtrpC F | ccctcgtctgaACTTAACGTTACTGAAATCATCAAACAG |
| TtrpC R | acggtatcgataagcttgatatcgcggccgcTCTAGAAAGAAGGATTACCTCTAAACAA |
| SucC F | CAGCCACCTCGTAACCCACCA |
| SucC R | GGTCGATCACGAAGCCGAGGT |
| SucD F | CCATGACATGATCCGCCTCCAG |
| SucD R | ATGATGACCACCTTGCACTCGC |
| MCL F | ccagcttctttcagcaATGTCTTTTAGATTGCAACCAG |
| MCL R | gatttcagtaacgttaagtTTAAGCAGAAATCATTTCA |
| 5flank F | agtgaattcgagctcggtaccGTAGAAAGTACTTGTCTGTTAGAATTAACGG |
| 5flank R | gatggactgctccttctaCTAATTAACCCTCACTAAAGGGAACA |
| MTKbox F | agTAGAAGGAGCAGTCCATCTGCG |
| MTKbox R | gTTACTTGTACAGCTCGTCCATGCC |
| HPH F | ggacgagctgtacaagtaaCAGAAGATGATATTGAAGGAGCATTT |
| HPH R | ctgcagtgatgcCTATTCCTTTGCCCTCGGACG |
| 3flank F | aggaatagGCATCACTGCAGGGACATTGT |
| 3flank R | tctagaggatccccgggtaccGTTCGAGAAGGCTACCTCTCTTACTAG |
| MTTA F | atccataccggtGGTGAAGATGAGGCTGAACGG |
| MTTA R | ggactgctccttctagcggccgcCTTGAAGCTGCCAACTCAACC |

**Table S7** RT-PCR Primers

| Name | Description |
| --- | --- |
| RT-TrCBH2-F | ATGCATCAACTACGCCGTCA |
| RT-TrCBH2-R | CCAGCCGTTGTAGTTAGCGA |
| RT-AsBGA-F | TCGACCAAATTGAGGCCCTC |
| RT-AsBGA-R | GTGTTGTTGCAGTTGGAGGC |
| RT-gh34-F | GGCAAGAGACGGCATTGTTC |
| RT-gh34-R | GAAGTAGGGGTAATCGGCCG |
| RT-cbh1-F | ACCACCATCGAACAGCACAT |
| RT-cbh1-R | CTGGGTGACAACGGTGAACT |
| RT-gh62-F | CTCCCCTCTTCTCAACGCTG |
| RT-gh62-R | CTTGACCCAGACGAAGGCAT |
| RT-MTK F | CAGCCACCTCGTAACCCACCA |
| RT-MTK R | GGTCGATCACGAAGCCGAGGT |
| RT-MCL F | CGCTGGTATTGCTCATGTTGA |
| RT-MCL R | TCTTGAGTACCACCAATTCCAG |

3.Optimized Sequence

**CAD**

ATGACCAAGCAGTCCGCCGACTCGAACGCCAAGTCGGGCGTGACCTCGGAAATATGCCACTGGGCCTCCAACCTTGCCACCGACGACATCCCCAGCGACGTGCTTGAGCGCGCCAAGTACCTTATCCTGGACGGTATCGCCTGCGCCTGGGTGGGTGCCCGCGTCCCCTGGTCGGAGAAGTATGTCCAGGCCACCATGTCGTTCGAGCCCCCCGGTGCGTGCAGGGTAATCGGCTACGGTCAGAAATTAGGTCCCGTGGCCGCCGCCATGACCAACTCCGCCTTCATCCAGGCCACCGAGTTGGACGACTACCACAGCGAGGCCCCCTTGCACAGCGCCAGCATCGTGCTTCCCGCCGTCTTCGCCGCCTCGGAGGTGTTGGCCGAGCAGGGCAAGACCATCTCGGGTATCGACGTGATCCTGGCCGCCATCGTGGGTTTCGAGTCGGGTCCCAGGATCGGCAAGGCCATCTACGGCTCGGACTTGCTGAACAACGGCTGGCACTGCGGCGCCGTCTACGGTGCCCCCGCCGGCGCCCTGGCCACCGGCAAGCTGTTGGGTCTTACCCCCGACAGCATGGAGGACGCCCTGGGTATCGCCTGCACCCAGGCCTGCGGTCTGATGTCGGCCCAGTACGGCGGCATGGTGAAGCGCGTCCAGCACGGTTTCGCCGCCCGCAACGGTCTTTTGGGCGGTCTTCTTGCCCACGGCGGTTACGAGGCCATGAAGGGTGTGCTTGAGCGCTCCTACGGTGGTTTCCTCAAGATGTTCACCAAGGGCAACGGTAGGGAGCCCCCCTACAAAGAAGAAGAGGTCGTGGCCGGTTTGGGCTCGTTCTGGCACACCTTCACCATCCGCATCAAGTTATACGCCTGCTGCGGTTTGGTCCACGGTCCCGTGGAGGCCATCGAGAACCTTCAGGGTCGATACCCCGAGTTGCTTAACAGGGCCAACCTCTCGAACATCAGGCACGTCCATGTGCAGCTTAGCACCGCCTCCAACTCGCACTGCGGCTGGATACCCGAAGAGAGGCCCATCTCCTCGATCGCCGGTCAGATGAGCGTGGCCTACATCCTTGCCGTGCAGTTGGTGGACCAGCAGTGCCTTTTGTCGCAGTTCTCCGAGTTCGACGACAACCTTGAGAGGCCCGAGGTGTGGGACTTGGCCAGGAAGGTGACCTCCTCGCAGAGCGAGGAATTTGACCAGGACGGTAACTGCTTGTCCGCCGGTAGGGTGCGCATCGAGTTCAACGACGGTAGCTCGATCACCGAGTCCGTGGAGAAGCCCTTGGGCGTCAAGGAACCCATGCCCAACGAGAGGATCTTGCACAAGTACAGGACCCTTGCCGGCTCCGTCACCGACGAGTCGCGCGTGAAAGAAATCGAGGACCTTGTGTTGGGTTTGGACAGGCTTACCGACATCAGCCCCCTTCTTGAGCTGTTGAACTGCCCCGTCAAGAGCCCCCTCGTCTGA

**TrCBH2**

ATGCTCGCCAAGTTCGCTGCCCTTGCGGCCCTTGTGGCCTCTGCCAACGCCCAAGCCTGCTCCTCCGTTTGGGGCCAGTGCGGCGGTCAAAACTGGTCAGGTCCCACCTGCTGCGCATCTGGATCTACTTGTGTTTATAGTAACGATTACTATTCCCAATGCCTCCCTGGCGCTGCTTCTTCCAGCAGTAGCACCAGAGCTGCTTCTACTACAAGTAGAGTTTCTCCTACTACTAGCAGAAGTTCCTCTGCCACTCCCCCCCCTGGCTCCACCACAACTAGGGTGCCCCCCGTCGGCAGCGGCACCGCCACCTACTCCGGCAACCCCTTCGTCGGCGTCACCCCCTGGGCCAACGCCTACTACGCCAGCGAGGTCAGCTCCCTCGCGATCCCCTCCCTGACCGGCGCCATGGCCACCGCCGCCGCCGCCGTCGCCAAGGTCCCCTCCTTCATGTGGCTCGATACCCTCGATAAGACCCCCCTCATGGAGCAGACCCTCGCCGACATCCGCACCGCCAACAAGAACGGCGGCAACTACGCCGGCCAGTTCGTCGTCTACGACCTCCCCGACCGCGACTGCGCCGCCCTGGCCTCCAACGGCGAGTACTCCATCGCCGACGGCGGCGTCGCTAAGTACAAGAACTACATCGACACCATTCGCCAGATCGTGGTCGAGTACTCCGACATCCGCACCCTGCTCGTCATCGAGCCCGACTCCCTGGCCAACCTCGTCACCAACCTCGGCACCCCCAAGTGCGCCAACGCCCAGAGCGCCTACCTGGAATGCATCAACTACGCCGTCACCCAGCTCAACCTCCCCAACGTCGCCATGTACCTCGACGCCGGCCACGCCGGCTGGCTGGGCTGGCCCGCCAACCAGGACCCCGCCGCCCAGCTCTTCGCCAACGTGTACAAGAACGCCAGCTCGCCCCGCGCCCTCCGCGGCCTCGCCACCAACGTCGCTAACTACAACGGCTGGAACATCACCTCCCCGCCCTCCTACACCCAGGGTAACGCCGTCTACAACGAGAAGCTCTACATCCACGCCATCGGCCCCCTCCTCGCCAACCACGGCTGGAGCAACGCCTTCTTTATCACCGACCAGGGCCGCTCCGGTAAACAGCCCACCGGCCAGCAGCAGTGGGGCGACTGGTGCAACGTCATCGGCACCGGCTTCGGCATCCGCCCCAGCGCCAACACCGGCGACAGCCTCCTCGACAGCTTCGTCTGGGTCAAGCCCGGCGGCGAGTGCGACGGCACCTCCGACAGCAGCGCCCCCAGGTTCGACTCCCACTGCGCCCTCCCCGACGCCCTCCAGCCCGCGCCCCAGGCCGGCGCCTGGTTCCAGGCCTACTTCGTCCAGCTCCTCACCAACGCCAACCCCTCCTTCCTCTGA

**AsBGA**

ATGCTCGCCAAGTTCGCTGCCCTTGCGGCCCTTGTGGCCTCTGCCAACGCCGACGAGCTGGCCTACTCCCCCCCCTACTACCCCTCCCCCTGGGCCAACGGCCAGGGCGACTGGGCCGAGGCCTACCAGAGGGCCGTCGACATCGTCTCCCAGATGACCCTGGCTGAGAAGGTCAACCTCACCACCGGCACCGGCTGGGAGCTCGAGCTGTGTGTCGGCCAGACGGGCGGCGTCCCCCGCCTGGGCATCCCTGGCATGTGCGCCCAGGACTCCCCCCTCGGCGTCCGCGACAGCGACTACAACAGCGCCTTCCCCGCCGGCGTCAACGTGGCCGCTACCTGGGACAAGAACCTCGCCTACCTCCGTGGCCAGGCCATGGGCCAGGAGTTCAGCGACAAGGGCGCCGACATCCAGCTCGGCCCGGCCGCGGGCCCCCTGGGCCGCTCCCCCGACGGCGGCCGTAACTGGGAGGGCTTCAGCCCCGACCCCGCCCTGTCGGGTGTTCTCTTCGCCGAGACGATCAAGGGCATCCAGGACGCCGGCGTCGTCGCGACCGCCAAGCACTACATCGCCTACGAGCAAGAGCACTTCAGGCAGGCGCCAGAGGCCCAGGGCTACGGCTTCAACATCACCGAGAGCCGCTCCGCTAACCTTGACGATAAGACCATGCATGAGCTCTACCTCTGGCCATTCGCCGACGCCATTAGGGCCGGCGCCGGTGCCGTCATGTGCTCCTACAACCAGATTAACAACTCGTACGGCTGCCAAAACTCCTACACCCTGAACAAGCTCCTCAAGGCCGAGCTCGGCTTCCAAGGCTTCGTCATGAGCGACTGGGCTGCCCACCACGCCGGCGTGTCCGGCGCCCTCGCCGGCCTTGACATGAGCATGCCCGGCGACGTGGACTACGACTCCGGCACCTCCTACTGGGGCACCAACCTCACCATCTCGGTCCTGAACGGCACCGCCCCACAGTGGCGCGTGGACGACATGGCCGTGCGCATCATGGCCGCCTACTACAAGGTGGGCCGCGACAGGCTCTGGACCCCCCCCAACTTCTCGTCTTGGACCCGCGACGAGTACGGCTTCAAGTACTACTACGTCTCCGAGGGTCCTTACGAGAAGGTGAACCAGTTCGTGAACGTCCAGCGGAACCATTCCGAGCTCATTCGCCGCATCGGCGCGGATAGCACCGTCCTCCTGAAGAACGATGGCGCCCTGCCCCTCACCGGCAAGGAGCGCCTGGTCGCCTTGATCGGCGAGGACGCCGGCAGCAACCCCTACGGCGCCAACGGCTGTAGCGACCGCGGCTGCGACAACGGCACCCTCGCTATGGGCTGGGGCAGCGGCACGGCCAACTTCCCCTACCTCGTCACCCCTGAGCAGGCCATCAGCAACGAGGTCTTGAAGAACAAGAACGGTGTGTTTACCGCCACCGATAACTGGGCCATCGACCAAATTGAGGCCCTCGCCAAGACCGCCTCCGTTAGCCTCGTCTTCGTCAACGCGGACAGCGGCGAGGGCTACATTAACGTCGACGGCAACCTCGGCGACCGCCGCAACCTCACGCTCTGGCGCAACGGCGACAACGTCATCAAGGCCGCTGCCTCCAACTGCAACAACACCATCGTGATCATCCATTCCGTGGGCCCCGTCCTCGTCAACGAGTGGTATGATAACCCTAACGTCACCGCCATCCTGTGGGGCGGCCTCCCCGGCCAGGAGTCCGGTAACAGCCTCGCCGACGTCCTCTACGGCCGGGTGAACCCTGGTGCCAAGTCCCCTTTCACCTGGGGGAAGACCCGCGAGGCCTACCAGGACTACCTCTACACCGAGCCGAACAACGGAAACGGCGCCCCCCAGGAGGATTTCGTTGAGGGTGTCTTTATCGACTACCGGGGCTTTGATAAGCGCAACGAGACCCCCATCTACGAGTTCGGCTACGGCTTGAGCTATACCACCTTCAACTACTCCAACCTCCAGGTCGAGGTTCTCTCGGCCCCCGCCTACGAGCCCGCCTCGGGCGAGACCGAGGCGGCCCCAACCTTCGGCGAGGTTGGCAACGCCTCGGACTACCTCTACCCCGATGGCCTCCAGAGGATCACGAAGTTCATCTACCCCTGGCTCAACTCGACCGACCTCGAGGCCAGCTCTGGCGACGCCTCCTACGGCCAAGACGCCTCGGATTACCTCCCCGAGGGCGCCACCGACGGCTCCGCCCAGCCGATCCTCCCGGCCGGCGGCGGCGCCGGTGGTAACCCCCGCCTCTACGACGAGCTGATCCGCGTCTCCGTCACCATCAAGAACACCGGCAAGGTCGCCGGCGACGAGGTCCCCCAGCTGTACGTCTCGCTCGGCGGCCCCAACGAGCCCAAGATCGTGCTCCGCCAGTTTGAGCGAATCACCCTCCAGCCGTCGAAGGAGACCCAGTGGAGCACCACCCTCACCCGCCGCGACCTCGCCAACTGGAACGTCGAGACCCAGGACTGGGAGATCACCTCCTACCCCAAGATGGTCTTCGCCGGCTCCAGCTCCCGGAAGCTCCCCCTCCGCGCCTCCCTCCCCACCGTCCACTGA

**MTTA**

ATGGATAGTAAGATCCAAACCAACGTCCCCCTCCCTAAGGCCCCATTGATTCAAAAGGCTAGAGGCAAGCGCACCAAGGGCATCCCCGCCCTCGTCGCCGGCGCCTGCGCCGGCGCAGTCGAGATCTCCATCACCTACCCCTTCGAGTCCGCCAAGACCCGCGCCCAGCTTAAGCGCCGCAACCATGACGTCGCCGCCATCAAACCCGGCATCCGCGGCTGGTACGCCGGCTACGGCGCCACCCTCGTCGGTACCACCGTCAAGGCCTCCGTCCAGTTCGCCTCCTTCAACATCTACCGCAGCGCCCTCAGCGGCCCCAACGGCGAGCTCAGCACCGGCGCCAGCGTCCTCGCCGGCTTCGGCGCCGGTGTCACCGAGGCCGTCCTCGCCGTCACCCCCGCCGAGGCCATCAAGACCAAGATCATCGACGCCCGCAAGGTCGGCAACGCCGAGCTCTCCACGACCTTCGGCGCCATCGCCGGCATCCTGCGCGACCGCGGCCCCCTCGGCTTCTTCTCCGCCGTCGGCCCCACCATCCTCCGCCAGTCCTCCAACGCCGCCGTTAAGTTCACCGTCTACAACGAGCTCATCGGCCTCGCCCGCAAGTACAGCAAGAACGGCGAGGACGTCCACCCCCTCGCCTCCACCCTCGTCGGCTCGGTCACCGGCGTCTGCTGCGCCTGGTCCACCCAGCCCCTCGACGTCATCAAGACCCGCATGCAGAGCCTCCAGGCCCGCCAGCTCTACGGCAACACCTTCAACTGCGTTAAGACCCTTTTGAGATCTGAGGGAATCGGTGTTTTCTGGTCCGGTGTCTGGTTCAGAACTGGTAGGTTGAGTTTGACATCTGCCATCATGTTCCCCGTCTACGAGAAGGTCTACAAGTTCCTCACCCAGCCTAACGGCGGCTCGGGCGGCGGCTCCGGCGGCGGCAGCGGC

**MTK**

SucC: ATGCGTGCCCCCGCTCTCCGTCGCAGCATCGCCACCACCGTTGTGCGCTGCAACGCCGAGACCAACATCCATGAGTACCAGGCCAAGGAGCTCCTCAAAACCTACGGCGTCCCTGTCCCGGATGGCGCCGTCGCCTACTCCGACGCCCAAGCCGCCAGCGTCGCCGAGGAGATCGGCGGCTCCCGCTGGGTCGTGAAGGCCCAGATCCACGCCGGCGGCCGCGGCAAGGCCGGCGGCGTTAAGGTCGCCCACAGCATCGAAGAGGTCCGACAGTACGCCGACGCCATGCTCGGCAGCCACCTCGTAACCCACCAGACTGGACCCGGCGGCTCCCTCGTGCAACGCCTCTGGGTCGAGCAGGCTTCTCACATCAAGAAGGAGTATTACCTCGGCTTCGTGATCGACCGGGGCAACCAGAGGATCACCCTTATCGCCTCCTCCGAGGGCGGCATGGAGATCGAGGAGGTGGCCAAGGAGACCCCCGAGAAGATCGTCAAGGAGGTCGTCGATCCCGCCATCGGCTTGCTGGACTTCCAGTGCCGGAAGGTGGCCACCGCGATCGGCTTGAAGGGCAAGCTGATGCCCCAGGCGGTCCGCCTTATGAAGGCCATCTACCGTTGCATGCGCGACAAGGACGCCTTGCAGGCCGAGATTAACCCCCTCGCCATCGTTGGCGAGAGTGACGAGAGCCTGATGGTCCTGGACGCCAAGTTCAACTTCGACGACAACGCCCTCTACCGGCAGCGCACCATCACCGAGATGCGTGACCTGGCTGAGGAGGACCCCAAGGAGGTGGAGGCCTCCGGCCACGGCCTCAACTACATTGCCCTCGACGGCAACATCGGCTGCATCGTCAACGGCGCCGGCCTCGCGATGGCCTCCCTCGACGCCATCACCCTCCACGGCGGCAGGCCGGCCAACTTCCTCGACGTCGGCGGCGGCGCGTCCCCCGAGAAGGTCACCAACGCCTGCCGCATCGTCCTTGAGGACCCCAACGTTCGCTGCATCCTCGTGAACATCTTCGCCGGCATCAACCGCTGCGATTGGATCGCCAAGGGCCTCATCCAGGCCTGCGATTCCCTCCAGATCAAGGTCCCCCTGATCGTCAGGCTCGCCGGCACCAACGTCGATGAGGGCCGCAAGATCCTGGCCGAGAGCGGCCTCTCCTTCATCACCGCCGAGAACCTCGACGACGCCGCCGCTAAGGCCGTCGCCATTGTGAAGGGC

Linker: GGCGGCTCGGGCGGCGGCTCCGGCGGCGGCAGCGGC

SucD: TCGGTCTTTGTCAACAAGCACTCCAAGGTCATCTTCCAGGGCTTCACCGGCGAGCATGCCACCTTCCACGCCAAGGACGCCATGAGGATGGGCACCCGCGTCGTCGGCGGCGTGACGCCTGGCAAGGGTGGCACCCGCCACCCCGACCCAGAGCTCGCGCACCTTCCGGTGTTCGACACCGTCGCAGAGGCCGTCGCCGCGACCGGTGCCGACGTGAGCGCCGTTTTCGTCCCCCCTCCCTTCAACGCCGACGCTCTGATGGAGGCCATCGACGCCGGCATCCGTGTCGCCGTGACGATCGCCGACGGCATCCCGGTCCATGACATGATCCGCCTCCAGCGCTACCGCGTGGGCAAGGACTCGATCGTCATCGGTCCCAACACCCCTGGCATCATCACCCCCGGCGAGTGCAAGGTGGGCATCATGCCCTCCCACATCTACAAGAAGGGCAACGTCGGCATCGTCAGTAGGTCCGGCACGCTGAACTACGAAGCCACCGAGCAGATGGCTGCCCTCGGCTTGGGCATCACCACCTCCGTCGGCATCGGCGGCGACCCAATCAACGGCACCGACTTCGTCACTGTCCTCAGGGCCTTCGAGGCCGACCCCGAGACCGAAATCGTTGTGATGATCGGCGAGATCGGCGGGCCCCAGGAGGTTGCTGCCGCCCGCTGGGCCAAGGAGAACATGACGAAGCCCGTGATCGGCTTCGTCGCCGGCCTCGCCGCCCCAACTGGCCGCCGCATGGGCCACGCCGGTGCCATTATCTCTTCTGAGGCCGACACCGCCGGCGCCAAGATGGACGCCATGGAGGCTCTGGGCCTGTACGTCGCCAGGAACCCTGCCCAGATCGGCCAGACCGTCCTCCGCGCGGCGCAAGAGCACGGCATCCGCTTC

**MCL**

ATGTCTTTTAGATTGCAACCAGCTCCACCAGCTAGACCTAACAGATGCCAATTGTTTGGTCCAGGTTCTAGACCAGCTTTGTTTGAAAAGATGGCTGCTTCTGCTGCTGATGTTATTAACCTTGATTTGGAAGATTCTGTTGCTCCTGATGATAAGGCCCAAGCTAGAGCTAACATTATTGAAGCTATTAACGGTTTGGATTGGGGTAGAAAGTACTTGTCTGTTAGAATTAACGGTTTGGATACTCCATTCTGGTACAGAGATGTTGTTGATCTTTTAGAACAAGCTGGTGACAGATTGGATCAAATTATGATTCCAAAGGTTGGTTGTGCTGCTGATGTTTACGCTGTTGATGCTTTGGTTACTGCCATTGAGAGAGCTAAGGGTAGAACTAAGCCATTGTCTTTTGAAGTTATTATTGAAAGCGCCGCTGGTATTGCTCATGTTGAAGAAATTGCTGCTTCTAGCCCTAGATTGCAAGCTATGTCTTTGGGTGCTGCTGACTTTGCTGCTTCTATGGGAATGCAAACTACTGGAATTGGTGGTACTCAAGAAAACTACTACATGTTGCATGACGGTCAAAAGCATTGGTCTGATCCATGGCATTGGGCTCAAGCTGCTATTGTTGCTGCTTGTAGAACTCATGGTATTTTGCCAGTTGATGGTCCATTCGGAGATTTCTCTGATGACGAAGGTTTTAGAGCTCAAGCTAGAAGATCTGCTACGCTGGGTATGGTTGGAAAGTGGGCTATTCATCCTAAGCAAGTTGCTTTGGCTAACGAGGTTTTTACTCCATCTGAGACTGCTGTTACTGAGGCTAGAGAAATTTTGGCTGCTATGGATGCTGCTAAAGCTAGAGGTGAAGGAGCTACTGTTTACAAGGGTAGATTGGTTGATATTGCTTCTATTAAGCAGGCTGAAGTTATTGTTAGACAAGCTGAAATGATTTCTGCTTAA
